# Actinoflavosides B–D, Flavonoid Type Glycosides from Tidal Mudflat-Derived Actinomyces

**DOI:** 10.3390/md20090565

**Published:** 2022-09-05

**Authors:** Hyeongju Jeong, Se Jin Jo, Munhyung Bae, Young Ran Kim, Kyuho Moon

**Affiliations:** 1 Research Institute of Pharmaceutical Sciences, College of Pharmacy, Chonnam National University, Gwangju 61186, Korea; 2 College of Pharmacy, Gachon University, Incheon 21936, Korea

**Keywords:** tidal mudflat, flavonoid, actinoflavoside, structural determination, secondary metabolite, antibacterial, immune modulator

## Abstract

Three new secondary metabolites, actinoflavosides B–D (**1**–**3**), were discovered in the culture broth of two actinomycete strains (JML48 and JMS33) that were isolated from tidal mudflat sediment in Muan, Republic of Korea. The planar structures of the actinoflavosides were elucidated by MS, UV, and NMR analyses. The stereochemistry of an aminosugar, 2,3,6-trideoxy-3-amino-ribopyranoside in the actinoflavosides was determined by *J*-based configuration analysis using values obtained from DQF-COSY experiments and modified Mosher’s method. Actinoflavosides B–D (**1**–**3**) displayed antibacterial activity against *Pseudomonas aeruginosa*, and actinoflavoside D (**3**) significantly increased IL-2 production in mouse splenocytes.

## 1. Introduction

Marine microorganisms are tremendously valuable as natural sources for chemical discovery due to the unique chemical structures and diverse bioactivities of their secondary metabolites, which have been developed as antibiotics, antifungal compounds, antitumor agents, and therapeutic agents [1,2]. Among natural products, flavonoids are a large and diverse group of bioactive polyphenols. Most flavonoids exhibit biological activities, such as anticancer [3], antioxidative [4], antimicrobial [5], and anti-inflammatory [6], and are therefore of great interest as natural compounds of pharmaceutical and chemical importance [7]. Flavonoids have long been known to exist in ferns and higher plants, but are relatively less common in microorganisms [8]. In 1997, a flavonoid, glycoside 7-{2,3,6-trideoxy-3-[3-(*R*)-hydroxy-2-(*R*)-methylbutanoic acid] amino-α-d-*ribo*-hexopyranosyl}-8-hydroxy-5-hydroxymethyl-2-phenyl-chroman-4-one, named actinoflavoside A, was isolated as a secondary metabolite from a marine-sourced *Streptomyces* sp. strain CNB-689 [9]. Since actinoflavoside A, flavonoid-type glycosides derived from marine actinomycetes have not been found.

In our search to discover new bioactive secondary metabolites from marine microorganisms, we isolated actinomycete strains from a tidal mudflat in Muan, Republic of Korea [10,11,12]. The tidal mudflat is greatly affected by extreme environmental changes, such as salinity, temperature, and water pressure, caused by tidal differences, and thus has high biodiversity, meeting our expectation of finding actinomycetes that produce various secondary metabolites. Metabolites of isolated strains were screened using liquid chromatography/mass spectrometry (LC/MS) analysis, and the *Streptomyces* sp. strains JML48 and JMS33 were identified to produce compounds containing a flavonoid moiety based on the characteristic ultraviolet (UV) and mass spectrometry (MS) data (UV *λ*_max_ = 285, 235, and 215 nm, [M + Na]^+^ *m/z* at 538, 518, and 480). Further scale-up of the culture of the strains was conducted, and three new compounds, namely actinoflavosides B, C, and D (1–3), were purified by high-performance liquid chromatography (HPLC). The planar structures were elucidated by one- and two-dimensional (1D and 2D) nuclear magnetic resonance (NMR) spectrometry and UV spectroscopy, and the absolute configurations of the compounds were determined by NMR analysis and Mosher’s method. Here, we report the isolation, structural determination, and biological activities of actinoflavosides B–D, unique metabolites from the *Streptomyces* sp. Moreover, from the in vitro biological activity assays, actinoflavoside D is suggested as a potential immunomodulatory agent.

## 2. Results and Discussion

### 2.1. Structural Elucidation

**Figure 1:**
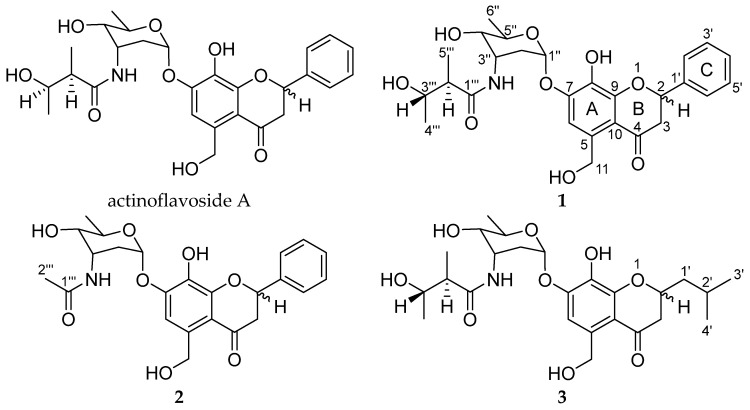
Structures of actinoflavosides A and B–D (**1**–**3**).

Actinoflavoside B (**1**) was obtained as a yellow oil, and its molecular formula was determined to be C_27_H_33_NO_9_ by high-resolution time-of-flight mass spectrometry (HR-TOF-MS) (*m/z* [M]^+^ 515.2172 calculated for C_27_H_33_NO_9_, 515.2155) in combination with ^1^H and ^13^C NMR data (Table 1). The ^1^H NMR spectrum of **1** in dimethyl sulfoxide (DMSO) showed six aromatic protons (δ_H_ 7.59 [2H], 7.43, 7.38 [2H], and 7.17); six protons attached to oxygen-bearing carbons (between 5.80 and 3.32 ppm); and three methyl group (δ_H_ 1.09, 1.06, and 1.03). The ^13^C NMR and HSQC data indicated that **1** has two carbonyl carbons (δ_C_ 192.3 and 174.8), 12 olefinic carbons between δ_C_ 151.5 and 106.5, six oxygenated carbons (δ_C_ 94.5, 78.6, 70.4, 68.3, 66.4, and 61.7), and three methyl groups (δ_C_ 21.2, 17.8, and 14.2). Based on the 1D and 2D NMR data analysis, including HSQC, COSY, HMBC, and TOCSY, compound **1** was determined to have three structural moieties; an aglycone bearing a 5-hydroxymethyl-7, 8-dihydroxyflavonoid; a 2,3,6-trideoxyaminosugar; and a 2-methyl-3-hydroxy-butyramide (Figure 1). First, the COSY correlations between H-2 (δ_H_ 5.61) and H_2_-3 (δ_H_ 3.16 and 2.79) established the connectivity of C-2 (δ_C_ 78.6) and C-3 (δ_C_ 44.7). The HMBC correlations from H-2 to C-4 (δ_C_ 192.3) and C-9 (δ_C_ 151.5), and from H_2_-3 to C-10 (δ_C_ 112.9) indicated the consecutive connectivity of C-2, C-3, C-4, C-9, and C-10. The HMBC correlations from the aromatic proton at H-6 (δ_H_ 7.17) to C-5 (δ_C_ 136.8), C-7 (δ_C_ 149.1), C-8 (δ_C_ 133.6), C-9, and C-10, and from aliphatic protons at H_2_-11 (δ_H_ 4.78) to C-5, C-6, and C-10 constructed the connectivity of C-5, C-6, C-7, C-8, C-9, and C-10. The remaining six-membered aromatic ring (C ring) was constructed by interpretation of the COSY correlations from H-2’ (δ_H_ 7.59) to H-6’ (δ_H_ 7.59). The HMBC correlations from H-2 to C-1’ (δ_C_ 139.1) confirmed the connectivity of C-2 and C-1’, establishing the aglycone moiety of **1**. The second partial structure was also determined by a combined analysis of the COSY, TOCSY, and HMBC data. The connectivity from C-1’’ (δ_C_ 94.5) to C-5’’ (δ_C_ 66.4) was confirmed by the COSY and TOCSY correlations from H-1’’ (δ_H_ 5.80) to H-5’’ (δ_H_ 3.79). The methyl group at C-6’’ (δ_C_ 17.8) was found to be located adjacent to C-5’’, based on the COSY correlations between H-5’’ and H-6’’ (δ_H_ 1.09), completing a 2,3,6-trideoxyaminosugar skeleton as a second partial structure of **1**. Thirdly, the aliphatic carbons C-2’’’ (δ_C_ 47.9) and C-3’’’ (δ_C_ 68.3) were linked by the COSY signals from H-2’’’ (δ_H_ 2.28) and H-3’’’ (δ_H_ 3.61). Further COSY correlations between methyl protons H_3_-4’’’ (δ_H_ 1.03) and H-3’’’ and other methyl protons H_3_-5’’’ (δ_H_ 1.06) to H-2’’’ placed the methyl group carbons C-4’’’ and C-5’’’ at C-3’’’ and C-2’’’, respectively, establishing 2-methyl-3-hydroxy-butyramide. Lastly, the HMBC correlations from H-1’’ to C-7 and from H-3’’and H-2’’’ to C-1’’’ (δ_C_ 174.8) constructed the connections between these three partial structures, finalizing the full planar structure of actinoflavoside B (**1**).

Actinoflavoside C (**2**) was isolated as a yellow oil, and its molecular formula was determined to be C_24_H_27_NO_8_ based on HR-TOF-MS (*m/z* [M]^+^ 457.1735 calculated for C_24_H_27_NO_8_, 457.1737) in combination with ^1^H and ^13^C NMR spectroscopic data (Table 1). The ^1^H NMR spectrum of (**2**) in DMSO showed features analogous to that of **1**, except for the terminal structure of the 2-methyl-3-hydroxy-butyramide moiety in actinoflavoside B (**1**). Instead of a 2-methyl-3-hydroxy-butyramide moiety, actinoflavoside C (**2**) has one methyl group H_3_-2’’’ (δ_H_ 1.91) connected to C-1’’’ (δ_C_ 169.6).

**Figure 2:**
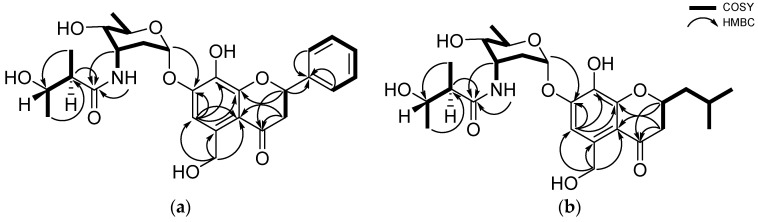
(**a**) Key ^1^H-^1^H COSY and HMBC correlations of actinoflavoside B (**1**); (**b**) Key correlations of actinoflavoside D (**3**).

Actinoflavoside D (**3**) was obtained as a red oil, and its molecular formula was assigned as C_25_H_37_NO_9_ by HR-TOF-MS (*m/z* [M]^+^ 495.2447 calculated for C_25_H_37_NO_9_, 495.2468) in combination with ^1^H and ^13^C NMR data (Table 1). Although **1** and **3** had similar ^1^H NMR spectra in DMSO, distinct differences in the chemical shifts of the aglycone moiety were found. Specifically, the chemical shift representing the aromatic ring (δ_H_ 7.59, 7.43, and 7.38) disappeared from the spectrum of (**3**), while signals for methine (δ_H_ 1.94), methylene (δ_H_ 1.79 and 1.47), and methyl groups (δ_H_ 0.92) were found. The emerged signals were connected by the COSY and TOCSY correlations from H_3_-4’ (δ_H_ 0.92) to H-3 (δ_H_ 2.62) (Figure 2).

**Figure 3:**
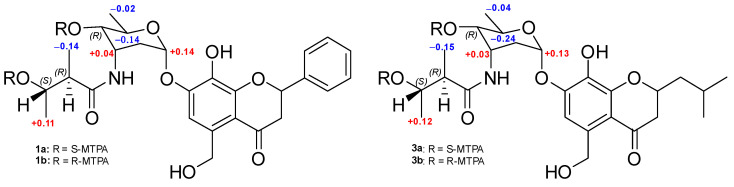
Δδ*_S-R_* values (ppm) obtained for the *S*- and *R*-MTPA esters (**1a**, **1b** and **3a**, **3b**).

The relative configuration of the aminosugar in actinoflavoside B (**1**) was determined based on its ^3^*J*_HH_ values, DQF-COSY, and ROESY NMR spectroscopic data. The magnitude of ^1^*J*_CH_ (171 Hz) between C-1’’ and H-1’’ established the anomeric carbon at C-1’’ as an α-configuration. The coupling constants of aminosugar protons measured by DQF-COSY (*J*_H3’’H4’’_ = 4.0 Hz and *J*_H4’’H5’’_ = 9.0 Hz) indicated that H-3’’ was in equatorial position and H-4’’, H-5’’ were in axial. The ROESY correlations further supported these assignments (Figure S31). To determine the relative configuration of 2-methyl-3-hydroxy-butyramide, *J*-based configuration analysis coupled with methyl decoupling experiments was conducted. The observed large coupling constants of (*J*_H2’’’H3’’’_ = 7.5 Hz) established the relative configurations of the stereogenic centers at C-2’’’ and C-3’’’ as *anti*. The absolute configuration of the secondary hydroxy groups at C-4’’-OH and C-3’’’-OH were determined by a modified version of Mosher’s method by utilizing *R*- and *S*-α-methoxy-α-(trifluoromethyl) phenylacetyl chloride (MTPA-Cl) and ^1^H NMR analysis. The calculated Δδ*_S-R_* values established the absolute configuration as 4’’*R* and 3’’’*S* (Figure 3). Thus, the structure of actinoflavoside B (**1**) was defined as the stereoisomer of actinoflavoside A. Actinoflavosides (**2**) and (**3**) were assigned to have the same stereochemistry as that determined for **1** based on a careful comparison of chemical shifts, DQF-COSY, and Mosher’s method. Stereogenic centers at C-2 of actinoflavosides B–D were confirmed as racemic.

### 2.2. Biological Evaluation

**Figure 4:**
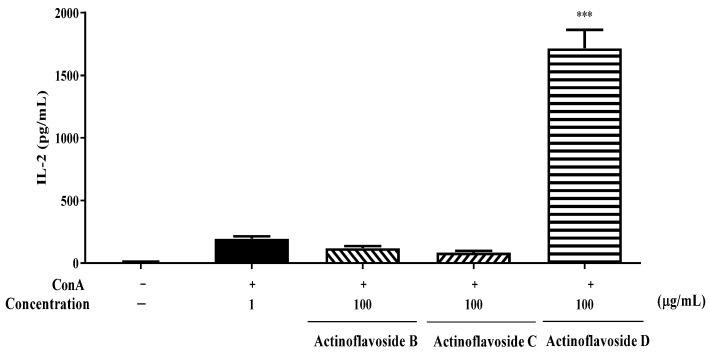
Effects of actinoflavosides on IL-2 production in splenocytes. Splenocytes were treated with actinoflavosides B, C, or D (100 µg/mL) in the presence of ConA for 72 h. IL-2 cytokine in the cell supernatant was measured by ELISA kit. The absorbance was read with an ELISA microplate reader at 450 nm. Data were expressed as mean ± SEM. *** *p* < 0.001 compared with the ConA treated group.

**Figure 5:**
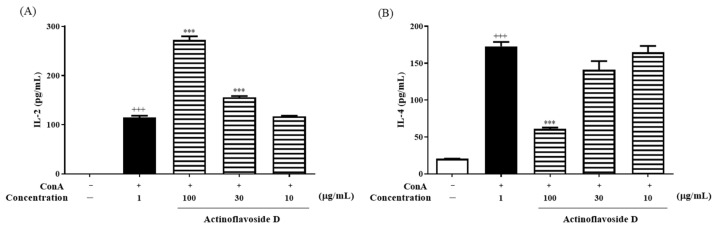
Effects of actinoflavoside D on IL-2 and IL-4 production in splenocytes. Splenocytes were treated with actinoflavoside D (10, 30, or 100 µg/mL) in the presence of ConA for 72 h. (**A**) IL-2 and (**B**) IL-4 cytokines in the cell supernatant were measured by ELISA kits. The absorbance was read at 450 nm using an ELISA microplate reader. Data were expressed as mean ± SEM. ^+++^ *p* < 0.001 compared with the non-treated group. *** *p* < 0.001 compared with the ConA treated group.

**Figure 6:**
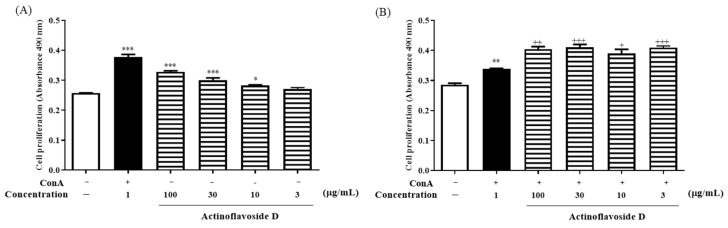
Effects of actinoflavoside D on splenocytes proliferation. Splenocytes isolated from BALB/c female mice (7 weeks) were treated with actinoflavoside D (3, 10, 30, or 100 µg/mL) in the (**A**) absence or (**B**) presence of ConA for 72 h, and then MTS reagents were added for 1 h. The absorbance was read at 490 nm using an ELISA microplate reader. Data were expressed as mean ± SEM. * *p* < 0.05, ** *p* < 0.01, *** *p* < 0.001 compared with the non-treated group. ^+^ *p* < 0.05, ^++^ *p* < 0.01, ^+++^ *p* < 0.001 compared with the ConA treated group.

In our endeavor to find new bioactive natural products, among a wide range of untargeted bioactivity tests, actinoflavosides B–D (**1**–**3**) showed antibacterial activities and immunomodulatory activity. First, actinoflavosides B–D were measured for growth inhibition activity against pathogenic bacteria, *Bacillus subtilis* ATCC 6051, *Pseudomonas aeruginosa* KCTC 22073, *Escherichia coli* ATCC 11775, and *Erwinia rhapontici* ATCC 29283. Actinoflavoside B and D inhibited the growth of *P. aeruginosa* with minimum inhibitory concentration (MIC) values of 0.29 and 0.30 mM, respectively. Actinoflavoside B showed antibacterial activity against *B. subtilis* with a MIC value of 0.14 mM. Additionally, to study the immunomodulatory activities of actinoflavosides B–D, interleukin-2 (IL-2) cytokine levels were measured in splenocytes isolated from mice. IL-2, a cytokine produced by Th1-type cells, is a T cell growth factor essential for T cell proliferation and differentiation [13,14]. Splenocytes were pretreated with actinoflavosides B–D (100 µg/mL) for 1 h and then concanavalin A (ConA; a T cell activator: 1 µg/mL) for 72 h. As shown in Figure 4, actinoflavoside D significantly increased the IL-2 cytokine production from splenocytes with ConA treatment. Next, we tested the effects of actinoflavoside D on the production of interleukin-4 (IL-4), a cytokine produced by Th2-type cells; this cytokine can produce many responses, some of which are associated with allergy and asthma [15]. Splenocytes were treated with actinoflavoside D (10, 30, or 100 µg/mL) and induced with ConA (1 µg/mL) for 72 h. IL-2 and IL-4 cytokines produced in the cell supernatants were detected by sandwich enzyme-linked immunosorbent assay (ELISA). As shown in Figure 5, actinoflavoside D significantly increased the IL-2 cytokine in ConA-induced splenocytes in a dose-dependent manner. By contrast, actinoflavoside D decreased the IL-4 cytokine production in ConA-induced splenocytes. Taken together, these results suggest that actinoflavoside D plays an important role in the T cell immune response by increasing primary T cell activation and IL-2 cytokine production without Th2-type cell activation. We investigated the effects of actinoflavoside D on splenocyte proliferation in the absence or presence of ConA. Splenocytes isolated from BALB/c mice spleens were treated with actinoflavoside D. As a result, the proliferation of splenocytes was significantly increased by actinoflavoside D (3, 10, 30, or 100 μg/mL) without (Figure 6A) or with (Figure 6B) ConA (1 µg/mL).

## 3. Materials and Methods

### 3.1. General Experimental Procedures

General rotations were measured by a PerkinElmer Model 343 plus polarimeter (Waltham, MA, USA) with a 1.0 cm cell. UV spectra were recorded using an Agilent Technologies 1260 Series Infinity II LC system (Agilent Technologies, Santa Clara, CA, USA). Infrared (IR) spectra were measured with a PerkinElmer spectrum 400 FT-IR and FT-NIR spectrometer (Waltham, MA, USA). ^1^H, ^13^C, and 2D NMR spectra were acquired on a Varian Unity INOVA 600 MHz spectrometer at the Korea Basic Science Institute (KBSI) at Gwangju and on a Bruker Avance II 800 MHz NMR spectrometer (Bruker, Billerica, MA, USA) at the KBSI at Ochang. Electrospray ionization (ESI) low-resolution LC/MS data were acquired on an Agilent G6125B MSD system coupled with an Agilent Technologies 1260 Series Infinity II LC system using a Phenomenex Luna reversed-phase C_18_ column (100 × 4.6 mm, 5 µm). High-Resolution TOF Mass Spectrum Field Desorption ion source data were acquired using a JMS-T200GC (Jeol, Akishima, Tokyo) at the Chonnam National University Cooperative Center for Research Facilities (CCRF). High-resolution electrospray ionization (HR-ESI) mass spectra were obtained using an Agilent Technologies 1290 Series HPLC coupled to an Agilent 6530 iFunnel Q-TOF LC/MS system (Agilent Technologies, Santa Clara, CA, USA).

### 3.2. Bacterial Isolation

The strains JML48 and JMS33 were isolated in August 2020 from a tidal mudflat sample in Muan, Republic of Korea. Dried sediment (2 g) was diluted in 4 mL of sterilized seawater, and the mixture was spread onto actinomycete isolation medium. The JML48 strain was isolated by culture on A1 agar medium (18 g agar, 25 mg cycloheximide, and 10 mg of nalidixic acid in 1 L seawater), and JMS33 was isolated by culture on TWYE agar medium (0.25 g of yeast extract, 0.5 g K_2_HPO_4_, 18 g agar, 25 mg cycloheximide, and 10 mg of nalidixic acid in 1 L seawater). The JML48 and JMS 33 strain were the most closely related to *Streptomyces althioticus* (99.9% identity, accession #LN864578), and *Streptomyces sanglieri* (99.0% identity, accession #AB735535) based on 16S rDNA sequencing analysis data.

### 3.3. Cultivation and Extraction

The JML48 was cultivated in 50 mL of YEME medium (3 g of yeast extract, 3 g of malt extract, 5 g peptone, 2 g soytone, and 10 g glucose in 1 L seawater) in a 100 mL Erlenmeyer flask. After the strains were cultivated at 25 °C for 3 days at 190 rpm on a rotary shaker, 3.5 mL of the culture was inoculated into a 500 mL Erlenmeyer flask containing 150 mL of YEME medium and shaken for 2 days. Then, 20 mL of the culture was inoculated into 1 L of YEME medium in a 2.5 L Ultra Yield flask. JML48 (8 L) was incubated at 25 °C and 190 rpm for 8 days. The entire culture was extracted with ethyl acetate (EtOAc), the substratum was separated using a separation funnel (capacity, 3 L), and the residual water in the EtOAc layer was removed by adding anhydrous Na_2_SO_4_. The extract was concentrated in vacuo to yield 3.5 g dry material. This procedure was repeated five times. JMS33 was cultivated and extracted in the same procedure on 4 days, to yield 2.8 g crude extract.

### 3.4. Isolation of Actinoflavosides

Each JML48, JMS33 dried material was fractionated over a C_18_ reversed-phase open column (YMC ODS-A-C_18_, 50 µm silica gel) with 100 mL each of 20%, 40%, 60%, 80%, and 100% MeOH in H_2_O and 1:1 MeOH/CH_2_Cl_2_. Actinoflavosides B–D were detected in the 60% and 80% MeOH/H_2_O fractions. The 60% and 80% fractions were separated by preparative reversed-phase HPLC (YMC-Pack ODS-A-C_18_ column, 5 µm, 250 × 20 mm) using an isocratic solvent system (33% CH_3_CN/H_2_O, UV detection at 254 nm, flow rate of 8 mL/min) t_R_ = 30 (42 mg), 35 (33 mg) min. Actinoflavosides B–D were further purified on a semi-preparative reversed-phase HPLC (YMC-Pack ODS-A-C_18_ column, 5 µm, 250 × 10 mm) using isocratic conditions (30% CH_3_CN/H_2_O, UV detection at 254 nm, flow rate: 2 mL/min). Actinoflaovsides B, C and D were collected as a pure compound at a retention time of 40 (15 mg), 36 (5.2 mg), and 48 (13 mg) min.

Actinoflaovside B (**1**): yellow oil, [α]_D_-79.9 (c 0.04, MeOH); UV (MeOH) λ_max_ (log ε) 215 (3.00) nm, 235 (3.25) nm, 285 (3.25) nm; IR (neat) ν_max_ 3374, 2933, 1601, 1300, 1063 cm^−1^, ^1^H and ^13^C NMR data see Table 1, HRMS (TOF) *m/z*: [M]^+^ 515.2172 (calcd for C_27_H_33_NO_9_, 515.2155).

Actinoflavoside C (**2**): yellow oil, [α]_D_-37.7 (c 0.02, MeOH); UV (MeOH) λ_max_ (log ε) 215 (3.08) nm, 235 (3.36) nm, 285 (3.36) nm; IR (neat) ν_max_ 3373, 2929, 1601, 1063 cm^−1^_,_ ^1^H and ^13^C NMR data see Table 1, HRMS (TOF) *m/z* [M]^+^ 457.1735 (calcd for C_24_H_27_NO_8_, 457.1737).

Actinoflavoside D (**3**): yellow oil, [α]_D_-173.97 (c 0.03, MeOH); UV (MeOH) λ_max_ (log ε) 215 (2.53) nm, 235 (2.66) nm, 285 (2.66) nm; IR (neat) ν_max_ 3376, 2933, 1601, 1294, 1061 cm^−1^_,_^1^H and ^13^C NMR data see Table 1, HRMS (TOF) *m/z* [M]^+^ 495.2447 (calcd for C_25_H_37_NO_9_, 495.2468).

### 3.5. MTPA Esterification of Actinoflavosides B and D

Samples of actinoflaovside B (**1**) were transferred to two 40 mL vials (3 mg of compound in each vial) and dried completely under high vacuum overnight. Then, 1 mL of anhydrous pyridine was added to each vial under N_2_ gas. The mixtures were stirred at room temperature for approximately 5 min. Then *R*- and *S*-α-methoxy-α-(trifluoromethyl) phenylacetyl chloride (MTPA-Cl) (30 μL) were each added into one of the two vials and the mixtures were stirred at 35 °C for 4 h. The products were then purified using a reversed-phase HPLC (YMC-Pack-C_8_ column, 5 μm, 250 × 10.0 mm) with a step gradient solvent system (50–100% CH_3_CN/H_2_O for 45 min, 100% CH_3_CN for 45 to 70 min, flow rate: 2 mL/min, detection: UV 280 nm), and both the *S*-MTPA ester of actinoflavoside B (**1a**) and the *R*-MTPA ester (**1b**) eluted at 51 min. The Δδ*_S-R_* values around the stereogenic centers were assigned by analyzing their ^1^H NMR and ^1^H-^1^H COSY NMR spectra. For actinoflaovside D (**3**), the same procedure was followed to yield **3a** and **3b**.

*S*-MTPA ester of actinoflavoside B (**1a**): ^1^H NMR (600 MHz, DMSO) δH 8.11 (NH, s), 7.59–7.38 (19H, overlapped), 5.84 (1H, dd, *J* = 6.0, 14.5), 5.75 (1H, m), 5.73 (1H, d, *J* = 14.5), 5.69 (1H, m), 5.17 (1H, t, *J* = 3.0), 5.12 (1H, dq, *J* = 12.5, 6.0) 4.40 (1H, s), 3.98 (1H, s), 3.34 (13H, s), 2.85–2.78 (1H, m), 2.58–2.53 (1H, m), 2.01 (1H, m), 1.88 (1H, m), 1.28 (3H, d, *J* = 6.0), 1.23 (3H, dd, *J* = 7.0, 5.5), 0.81 (3H, d, *J* = 7.0).

*R*-MTPA ester of actinoflavoside B (**1b**): ^1^H NMR (600 MHz, DMSO) δH 7.86 (NH, s), 7.58–7.40 (15H, overlapped), 7.33 (2H, m), 7.15–7.00 (3H, m), 5.88 (1H, t, *J* = 5.3), 5.82 (1H, s), 5.618 (1H, s), 5.14 (1H, m), 4.36 (1H, s), 4.21 (1H, dd, *J* = 7.0, 3.0), 3.36–3.30 (15H, s), 2.90 (1H, m), 2.83 (1H, m), 2.61 (1H, m), 2.48–2.45 (1H, m), 2.38 (1H, m) 1.97 (1H, s), 1.85 (1H, m), 1.25 (3H, m), 1.17 (3H, *J* = 6.0, 1.5), 0.95 (3H, *J* = 7.0).

*S*-MTPA ester of actinoflavoside D (**3a**): ^1^H NMR (600 MHz, DMSO) δH 8.091 (NH, s), 7.53–7.39 (22H, overlapped) 7.01 (1H, s), 5.79 (1H, d, *J* = 14.0), 5.74 (1H, m), 5.70 (1H, m), 5.17 (1H, t, *J* = 3.5), 5.11 (1H, dq, *J* = 11.5, 6.5), 4.62 (1H, m), 4.40 (1H, m), 3.98 (1H, dd, *J* = 7.0, 3.5), 3.37 (10H, s) 2.77 (1H, dd, *J* = 17.0, 4.0), 2.71 (1H, dd, *J* = 17.0, 4.0), 2.55 (1H, m), 2.02 (1H, m), 1.88 (1H, m), 1.64 (2H, overlapped), 1.42 (1H, m), 1.28 (3H, dd, *J* = 6.0, 2.0), 1.22 (3H, dd, *J* = 6.0, 2.0), 0.85–0.73 (6H, overlapped).

*R*-MTPA ester of actinoflavoside D (**3b**): ^1^H NMR (600 MHz, DMSO) δH 7.84 (NH, s), 7.53 (17H, overlapped), 6.98 (1H, d, *J* = 13.5), 5.80 (2H, m), 5.77 (2H, dt, *J* = 14.5, 9.0), 5.61 (1H, s), 5.13 (2H, m), 4.61 (1H, s), 4.36 (1H, s), 4.21 (1H, m), 3.38 (8H, s), 2.74 (2H, m), 2.47 (1H, m), 1.97 (1H, s), 1.85 (1H, m), 1.25 (3H, dd, *J* = 14.5, 9.0), 1.16 (3H, dd, *J* = 6.0, 4.0), 0.96 (3H, *J* = 7.0, 1.5), 0.85–0.71 (6H, overlapped).

### 3.6. Antibacterial Activity Assay

Gram-positive bacteria (*B. subtilis* ATCC 6051) and Gram-negative bacteria (*P. aeruginosa* KCTC 22073, *E. coli* ATCC 11775, and *Er. rhapontici* ATCC 29283) were cultured on Luria-Bertani agar (LB). After incubation at 30 °C overnight, the cells were cultured in LB broth at 30 °C for 24 h, and the harvested microbial cells were inoculated into Mueller-Hilton broth with an initial optical density (OD_600_) value of 0.0008. The compound solutions were prepared in DMSO. Each solution was diluted with Mueller-Hilton broth to generate serial two-fold dilutions in the range of 200 to 0.8 μg/mL. The plates were incubated at 30 °C for 20 h, and the OD values were observed at 600 nm. Gentamicin was used as a reference compound.

### 3.7. Immunomodulatory Activity Assay

Seven-week-old BALB/c female mice (Damool Science, Daejeon, Korea) were maintained in a pathogen-free animal room with controlled temperature and a 12 h light/dark cycle. All animal procedures followed the guidelines of the animal care and use committee of Chonnam National University (CNU IACUC-YB-2022-01). Mice were sacrificed by cervical dislocation. A single-cell suspension was prepared from the isolated spleen using a 50 μm mesh strainer (BD Falcon, San Diego, CA, USA). The cell pellet was thoroughly resuspended in red blood cell lysis buffer (Biolegend, San Diego, CA, USA) at room temperature for 5 min. Splenocytes were suspended in RPMI media and induced with ConA (1 μg/mL), a mitogen that non-specifically activates T-cells. Splenocytes were seeded in 48-well plates (SPL life sciences Co., Pocheon, GG, Korea) at 2 × 10^5^ cells/well. The cells were treated with actinoflavosides B, C, or D (10, 30, or 100 µg/mL) and ConA (1 µg/mL) for 72 h. IL-2 and IL-4 cytokines in the cell supernatants were measured using ELISA kits (R&D system, Minneapolis, MN, USA). The absorbance was read at 450 nm using an ELISA microplate reader (ELx808) (Bio Tek Instruments, Inc., Winooski, VT, USA). Mouse splenocytes were seeded in 96-well plates (SPL life sciences Co., Pocheon, GG, Korea) at 1 × 10^5^ cells/well. The cells were treated with actinoflavoside D (3, 10, 30, or 100 µg/mL) in the absence or presence of ConA for 72 h. Cell proliferation was assayed using 3-(4,5-dimethylthiazol-2-yl)-5-(3-carboxy-methoxyphenyl)-2-(4-sulfophenyl)-2H-tetrazolium (MTS) reagents according to the manufacturer’s instructions. The absorbance was read at 490 nm using an ELISA microplate reader (ELx808). All experiments were repeated more than three times on other days. The results were expressed as the mean ± standard error of the mean (SEM), unless otherwise stated. Statistical differences were evaluated using one-way analysis of variance (ANOVA) for multigroup comparisons, followed by a Tukey’s post hoc test, with *p* < 0.05 being considered statistically significant.

## 4. Conclusions

Actinoflavosides B–D, flavonoid-type glucosides, which have rarely been reported in marine actinobacteria, were discovered by LC/MS-based chemical screening of tidal mudflat-derived bacteria. Spectroscopic analysis (including 1D and 2D NMR spectroscopy) and chemical derivatization based on modified Mosher’s method revealed that the structures of the actinoflavosides were composed of an aglycone flavonoid backbone and an amino sugar moiety. Since the discovery of actinoflavoside A in 1997, any derivatives or similar metabolites have not been found for 25 years. Especially, actinoflavoside D has isobutyl alkylated chromone structure modified from a flavonoid structure, which is a very unique structure that has not been reported in natural products derived from microorganisms and plants. This study not only draws attention to the discovery of natural products after a very long time but is also important in reporting the new biological activity of the metabolites. In recent medicine, which emphasizes the treatment and prevention of infectious diseases worldwide, our research on marine microbial natural products suggests an effort to discover immunomodulatory therapeutic compounds.

## 5. Patents

This section is not mandatory but may be added if there are patents resulting from the work reported in this manuscript.

## Figures and Tables

**Table 1:** ^1^H and ^13^C NMR spectroscopic data for actinoflavosides B–D (**1**–**3**) in DMSO-*d_6_*.

	Actinoflavoside B *		Actinoflavoside C *		Actinoflavoside D *	
Position	δ_C_, Type	δ_H_, Mult (*J* in Hz)	δ_C_, Type	δ_H_, Mult (*J* in Hz)	δ_C_, Type	δ_H_, Mult (*J* in Hz)
2	78.6, CH	5.61, m	78.5, CH	5.6, m	75.8, CH	4.51, m
3	44.7, CH_2_	3.16, 2.79, m	44.6, CH_2_	3.15, 2.81, m	43.8 CH_2_	2.62, m
4	192.3, C		192.2, C		191.0, C	
5	136.8, C		136.9, C		136.6, C	
6	106.5, CH	7.17, s	106.3, CH	7.15, s	106.6, CH	7.10, s
7	149.1, C		149.1, C		148.8, C	
8	133.6, C		133.5, C		133.6, C	
9	151.5, C		151.6, C		151.0, C	
10	112.9, C		112.8, C		113.0, C	
11	61.7, CH_2_	4.78, s	61.6, CH_2_	4.77, s	61.6, CH_2_	4.74, s
1’	139.1, C		139.2, C		43.1, CH_2_	1.79, 1.47, m
2’, 6’	126.7, CH	7.59, m	126.7, CH	7.58, m	23.5, CH	1.94, m
3’, 5’	128.4, CH	7.38, m	128.3, CH	7.38, m	(2’ in **3**)22.3, CH_3_(3’ in **3**)	0.92, m
4’	128.5, CH	7.43, m	128.4, CH	7.44, m	22.8, CH_3_	0.92, m
1’’	94.5, CH	5.80, t (3.0)	94.3, CH	5.80, m	94.8, CH	5.77, t (3.0)
2’’	32.9, CH_2_	2.08, 1.99, m	32.7, CH_2_	2.03, 1.90, m	32.9, CH_2_	2.07, 1.98, m
3	44.8, CH	4.34, dt (8.5, 4.0)	45.4, CH	4.29, dt (8.5, 4.0)	44.8, CH	4.33, m
4’’	70.4, CH	3.32, dd (9.0, 4.0)	70.6, CH	3.30, dd (9.0, 4.0)	70.3, CH	3.31, m
5	66.4, CH	3.79, m	66.4, CH	3.76, m	66.5, CH	3.81, m
6’’	17.8, CH_3_	1.09, d (6.0)	17.9 CH_3_	1.07, d (6.0)	17.8, CH_3_	1.07, overlapped
NH		7.61, m		7.85, m		7.6, m
1’’’	174.8, C		169.6, C		171.0, C	
2’’’	47.9, CH	2.28, m	23.2, CH_3_	1.91, s	47.3, CH	2.30, m
3’’’	68.3, CH	3.61, dq (12.5, 6.0)			68.3, CH	3.63, dq (12.5, 6.0)
4’’’	21.2, CH_3_	1.03, d (6.0)			21.0, CH_3_	1.02, d (6.0)
5’’’	14.2, CH_3_	1.06, d (7.0)			14.2, CH_3_	1.08, overlapped

* ^1^H and ^13^C NMR data were recorded at 600 and 150 MHz, respectively.

## Data Availability

All data is contained within this article and Supplementary Materials.

## Supplementary Materials

The following supporting information can be downloaded at: https://www.mdpi.com/article/10.3390/md20090565/s1, Figures S1–S20: NMR spectra of actinoflavosides; Figure S21: Methyl decoupling ^1^H NMR spectrum of actinoflavoside B (**1**); Figure S22: DQF-COSY NMR spectrum of actinoflavoside B (**1**); Figures S23–S30: NMR spectra of MTPA ester for actinoflavosides; Figure S31: Key ROESY correlations of the 2, 3, 6-trideoxyaminosugar of acitnoflavoside B (**1**); Figures S32 and S33: 16 S rDNA sequence data of strains; Figure S34: UV spectra of actinoflavosides; Figures S35–S37: HR-TOF-MS data of actinoflavosides; Figures S38–S40: IR spectra of actinoflavosides; Figures S41 and S42: Decoupling ^1^H NMR spectra of actinoflavoside B (**1**); Table S1: Minimum inhibitory concentration (MIC) of (**1**–**3**) against Gram-positive and Gram negative bacterial strains.
