# Supplementary material for: Actinoflavosides B–D, Flavonoid Type Glycosides from Tidal Mudflat-Derived Actinomyces
Source: Mar Drugs. 2022 Sep 5;20(9):565. doi: 10.3390/md20090565 (PMC9503743; doi:10.3390/md20090565)
Supplement: Supplementary file 1 [file marinedrugs-20-00565-s001.zip › marinedrugs-1886866-supplementary.pdf]

Supplementary Material for

Actinoflavosides B-D, novel flavonoid type glycosides from tidal mudflat-derived actinomycete

Hyeongju Jeong[1,†], Se Jin Jo[1,†], Munhyung Bae[2],Young Ran Kim[1,*]and Kyuho Moon[1,*]

1 College of Pharmacy, Research Institute of Pharmaceutical Sciences, Chonnam National University, Gwangju, South Korea; 217843@jnu.ac.kr (H.J.); 216446@jnu.ac.kr (S.J.J);

2 College of Pharmacy, Gachon University, Incheon, South Korea; baemoon89@gachon.ac.kr (M.B.)

† These authors contributed equally to this work.

Corresponding Author

* Correspondence: kimyr@chonnam.ac.kr (Y.R.K.), khmoon@jnu.ac.kr (K.M.)

Table of contents

Figure S1. ^{1}H NMR spectrum of actinoflavoside B (**1**) at 600 MHz in DMSO.

Figure S2. ^{13}C NMR spectrum of actinoflavoside B (**1**) at 150 MHz in DMSO.

Figure S3. COSY NMR spectrum of actinoflavoside B (**1**) in DMSO.

Figure S4. HSQC NMR spectrum of actinoflavoside B (**1**) in DMSO.

Figure S5. HMBC NMR spectrum of actinoflavoside B (**1**) in DMSO.

Figure S6. ROESY NMR spectrum of actinoflavoside B (**1**) in DMSO.

Figure S7. TOCSY NMR spectrum of actinoflavoside B (**1**) in DMSO.

Figure S8. ^{1}H NMR spectrum of actinoflavoside C (**2**) at 600 MHz in DMSO.

Figure S9. ^{13}C NMR spectrum of actinoflavoside C (**2**) at 150 MHz in DMSO.

Figure S10. COSY NMR spectrum of actinoflavoside C (**2**) in DMSO.

Figure S11. HSQC NMR spectrum of actinoflavoside C (**2**) in DMSO.

Figure S12. HMBC NMR spectrum of actinoflavoside C (**2**) in DMSO.

Figure S13. ROESY NMR spectrum of actinoflavoside C (**2**) in DMSO.

Figure S14. ^{1}H NMR spectrum of actinoflavoside D (**3**) at 600 MHz in DMSO.

Figure S15. ^{13}C NMR spectrum of actinoflavoside D (**3**) at 150 MHz in DMSO.

Figure S16. COSY NMR spectrum of actinoflavoside D (**3**) in DMSO.

Figure S17. HSQC NMR spectrum of actinoflavoside D (**3**) in DMSO.

Figure S18. HMBC NMR spectrum of actinoflavoside D (**3**) in DMSO.

Figure S19. ROESY NMR spectrum of actinoflavoside D (**3**) in DMSO.

Figure S20. TOCSY NMR spectrum of actinoflavoside D (**3**) in DMSO.

Figure S21. Methyl decoupling ^{1}H NMR spectrum of actinoflavoside B (**1**) at 800 MHz in DMSO

Figure S22. DQF-COSY NMR spectrum of actinoflavoside B (**1**) at 800 MHz in DMSO

Figure S23. ^{1}H NMR spectrum of S-MTPA ester (**1a**) for actinoflavoside B (**1**) at 600 MHz in DMSO.

Figure S24. COSY NMR spectrum of S-MTPA ester (**1a**) for actinoflavoside B (**1**) at 600 MHz in DMSO.

Figure S25. ^{1}H NMR spectrum of R-MTPA ester (**1b**) for actinoflavoside B (**1**) at 600 MHz in DMSO.

Figure S26. COSY NMR spectrum of R-MTPA ester (**1b**) for actinoflavoside B (**1**) at 600 MHz in DMSO.

Figure S27. ^{1}H NMR spectrum of S-MTPA ester (**3a**) for actinoflavoside D (**3**) at 600 MHz in DMSO.

Figure S28. COSY NMR spectrum of S-MTPA ester (**3a**) for actinoflavoside D (**3**) at 600 MHz in DMSO.

Figure S29. ^{1}H NMR spectrum of R-MTPA ester (**3b**) for actinoflavoside D (**3**) at 600 MHz in DMSO.

Figure S30. COSY NMR spectrum of R-MTPA ester (**3a**) for actinoflavoside D (**3**) at 600 MHz in DMSO.

Figure S31. Key ROESY correlations of the 2, 3, 6-trideoxyaminosugar of acitnoflavoside B (**1**).

Figure S32. 16S rDNA sequence data of JML48.

Figure S33. 16S rDNA sequence data of JMS33.

Figure S34. UV spectrum of actinoflavoside B-D (**1-3**).

Figure S35. HR-TOF-MS data of actinoflavosdie B (**1**).

Figure S36. HR-TOF-MS data of actinoflavosdie C (**2**).

Figure S37. HR-TOF-MS data of actinoflavosdie D (**3**).

Figure S38. IR spectrum of actinoflavosdie B (**1**).

Figure S39. IR spectrum of actinoflavosdie C (**2**).

Figure S40. IR spectrum of actinoflavosdie D (**3**).

Figure S41. H-4′′′ decoupling ^{1}H NMR spectrum (800 MHz) of actinoflavoside B (**1**) in DMSO.

Figure S42. H-5′′′ decoupling ^{1}H NMR spectrum (800 MHz) of actinoflavoside B (**1**) in DMSO.

Table S1. Minimum inhibitory concentration (MIC) of **1-3** against Gram-positive and Gram-negative bacterial strains.

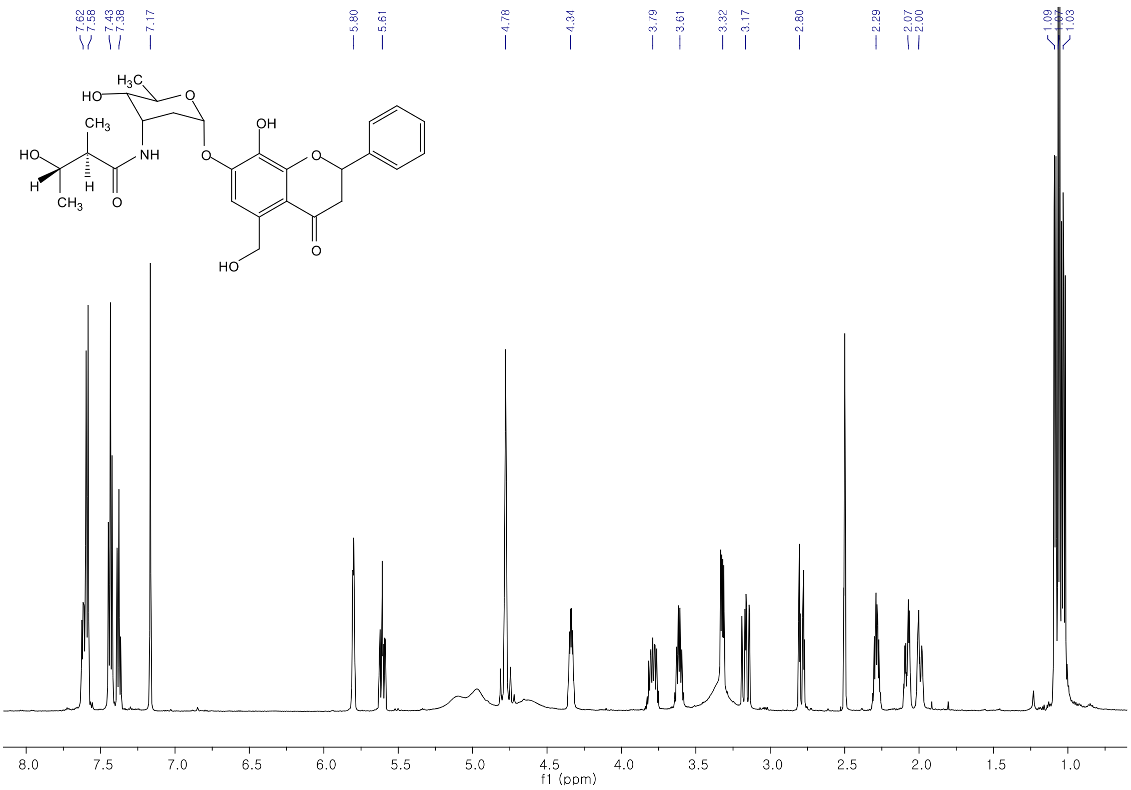

Figure S1. ^{1}H NMR spectrum of actinoflavoside B (**1**) at 600 MHz in DMSO.

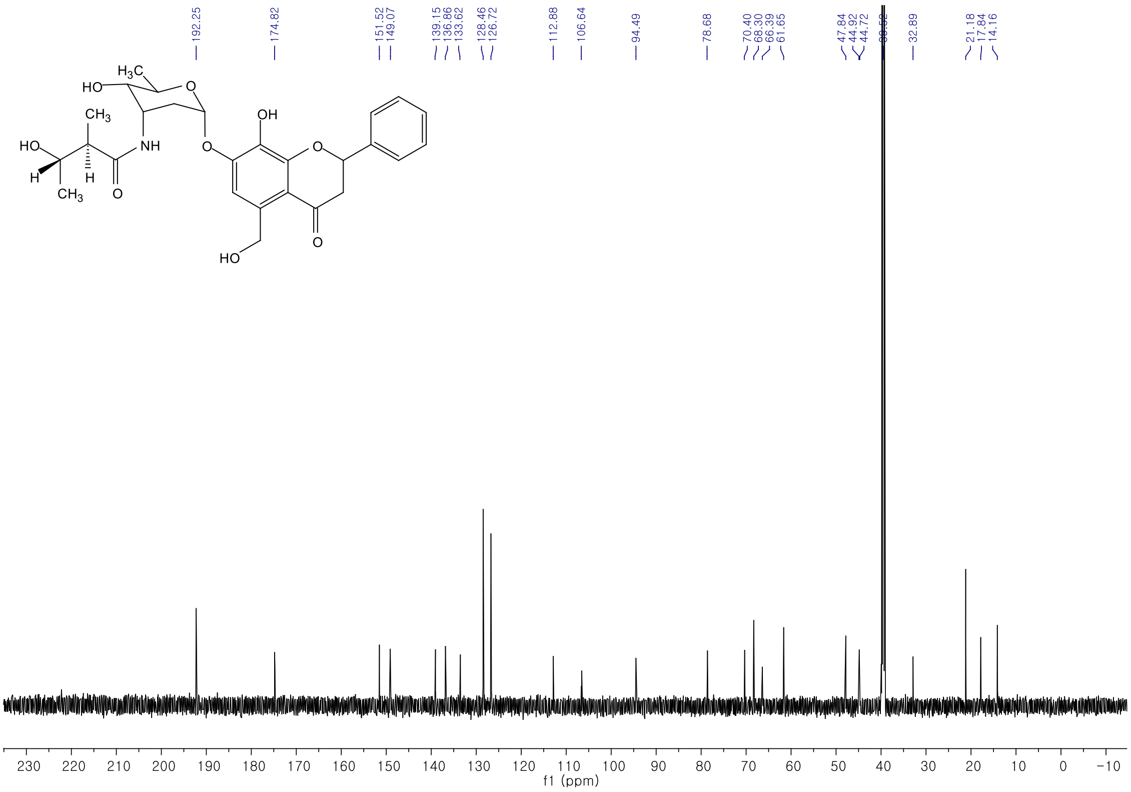

Figure S2. ^{13}C NMR spectrum of actinoflavoside B (**1**) at 150 MHz in DMSO.

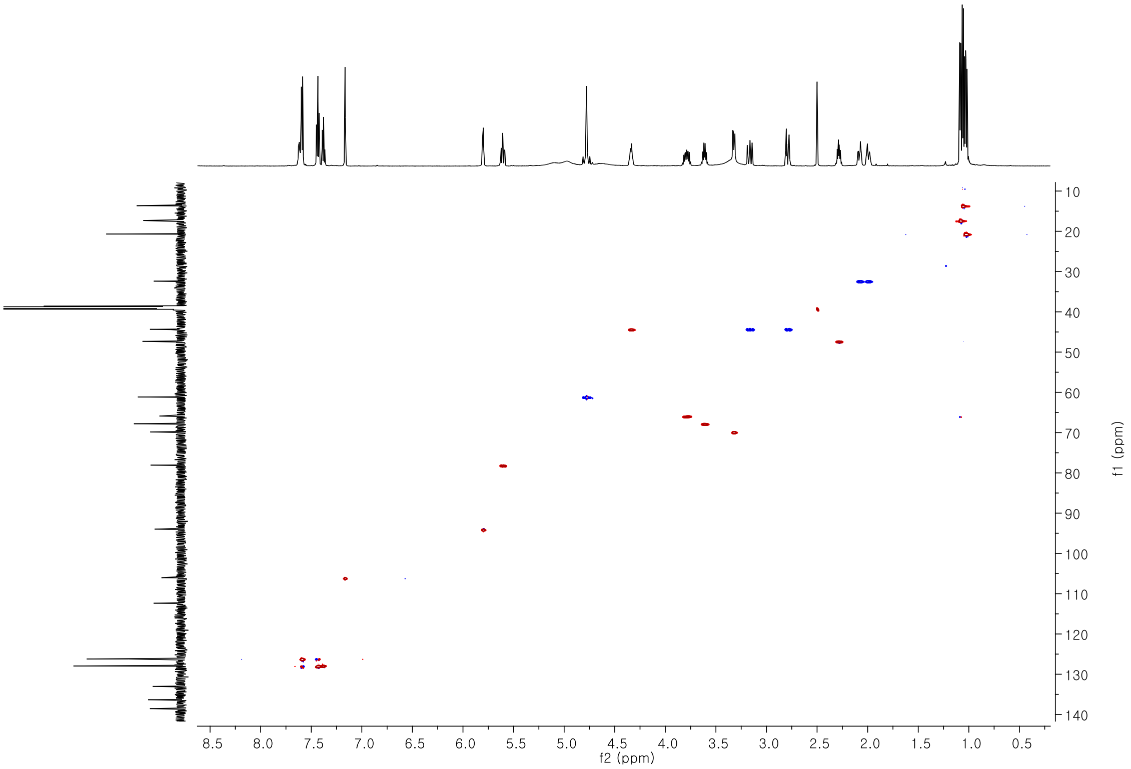

Figure S3. COSY NMR spectrum of actinoflavoside B (**1**) in DMSO.

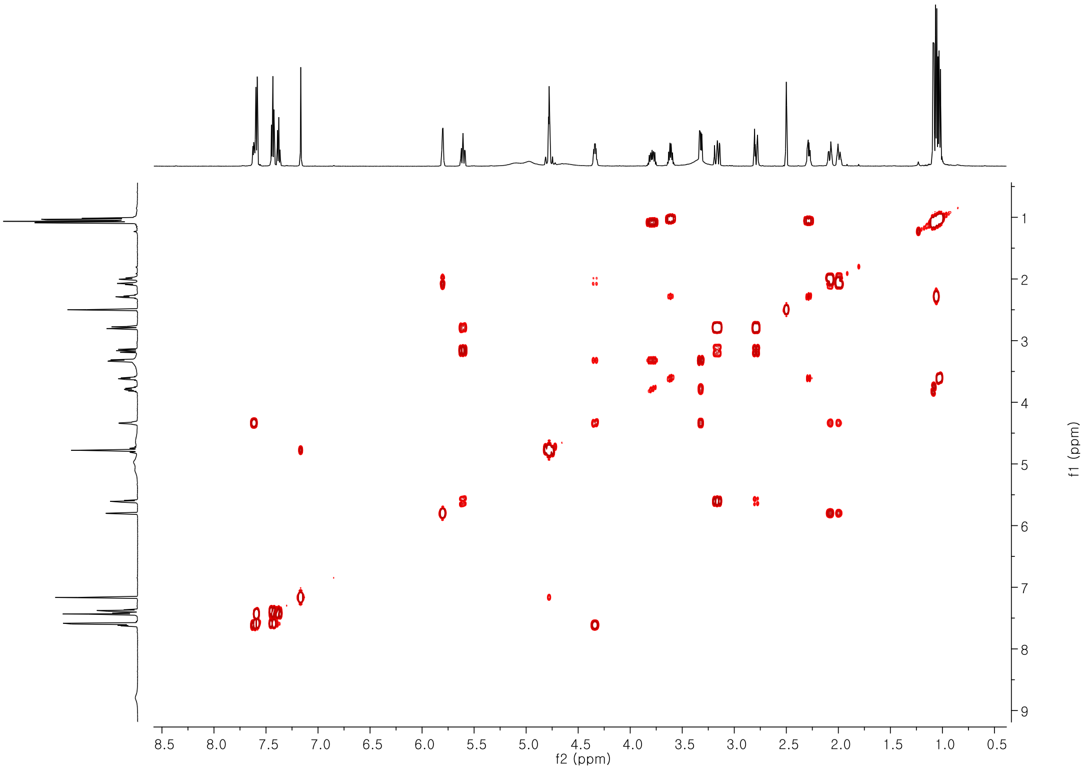

Figure S4. HSQC NMR spectrum of actinoflavoside B (**1**) in DMSO.

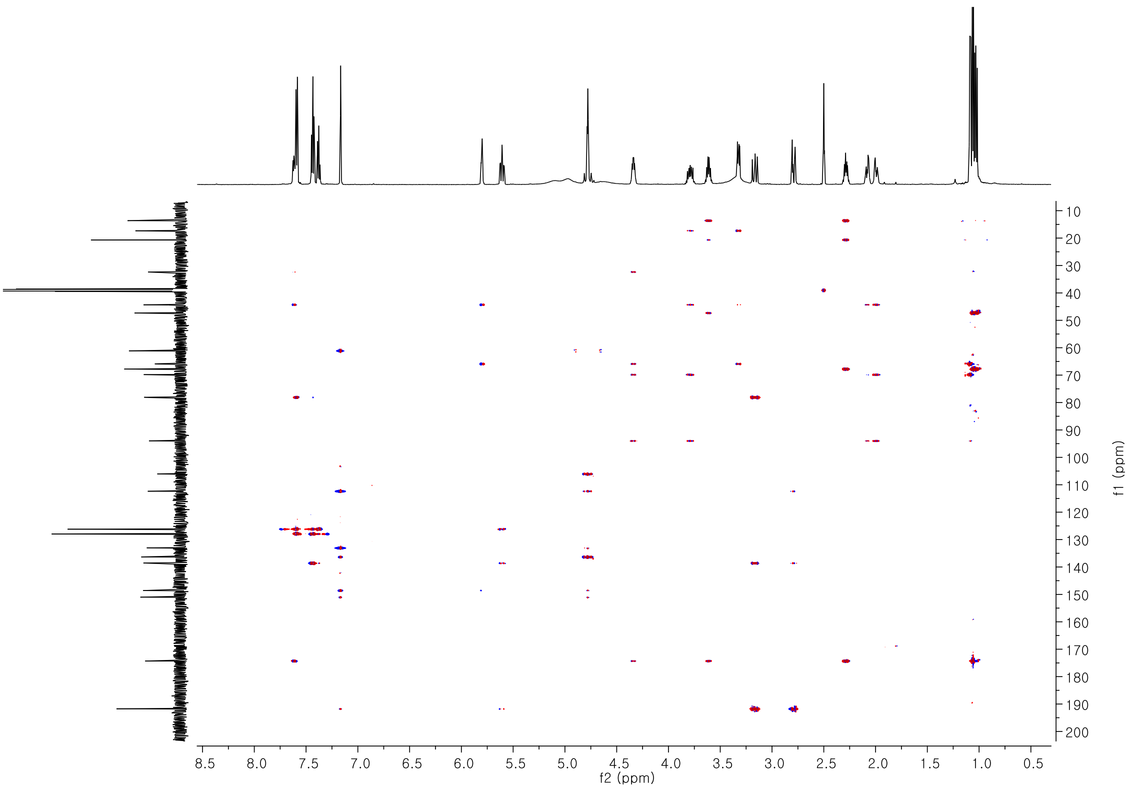

Figure S5. HMBC NMR spectrum of actinoflavoside B (**1**) in DMSO.

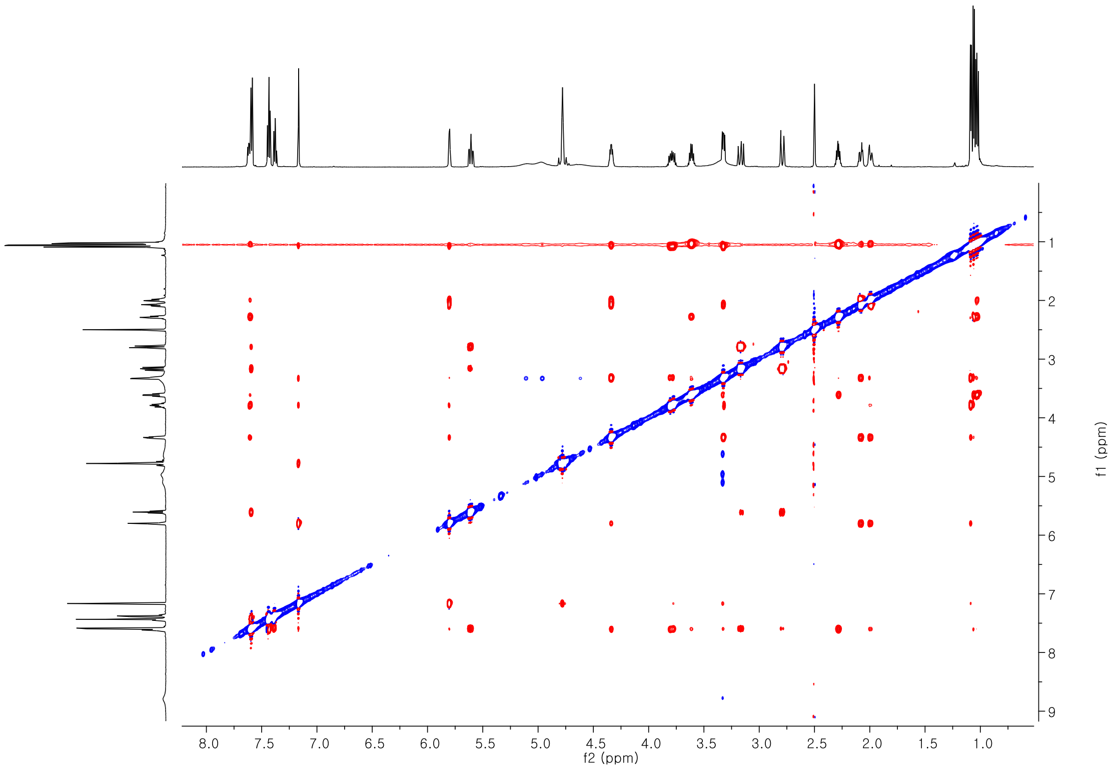

Figure S6. ROESY NMR spectrum of actinoflavoside B (**1**) in DMSO.

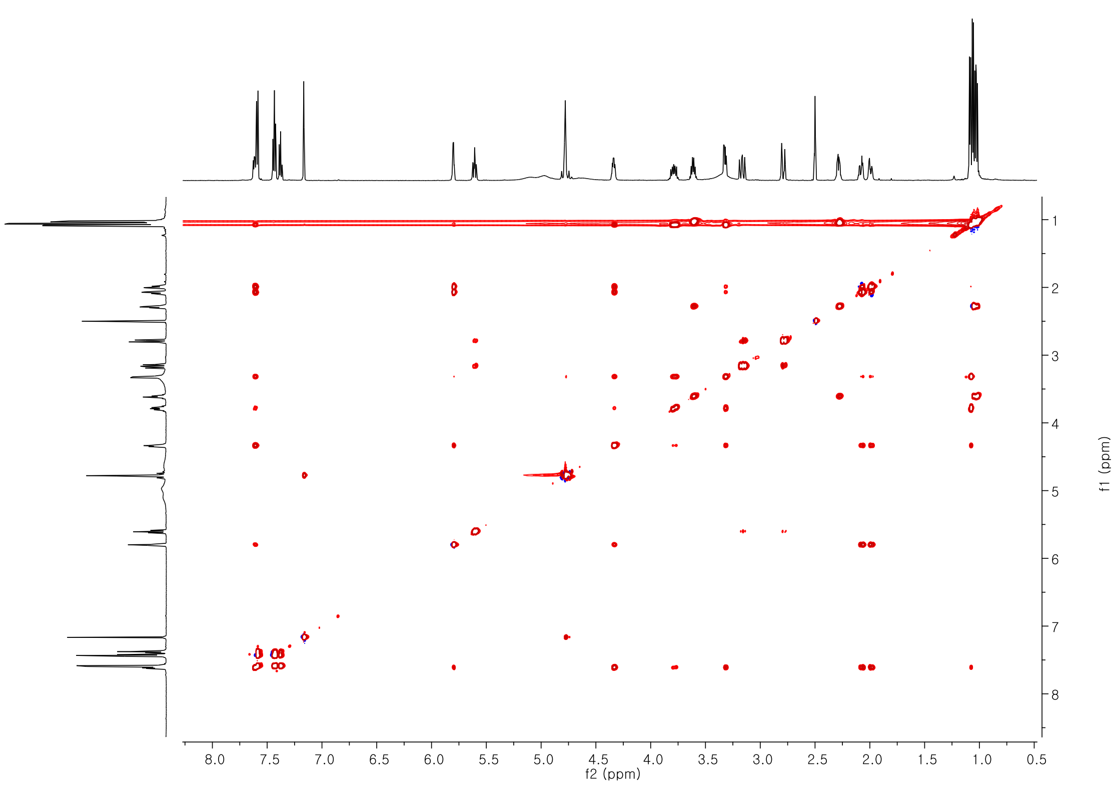

Figure S7. TOCSY NMR spectrum of actinoflavoside B (**1**) in DMSO.

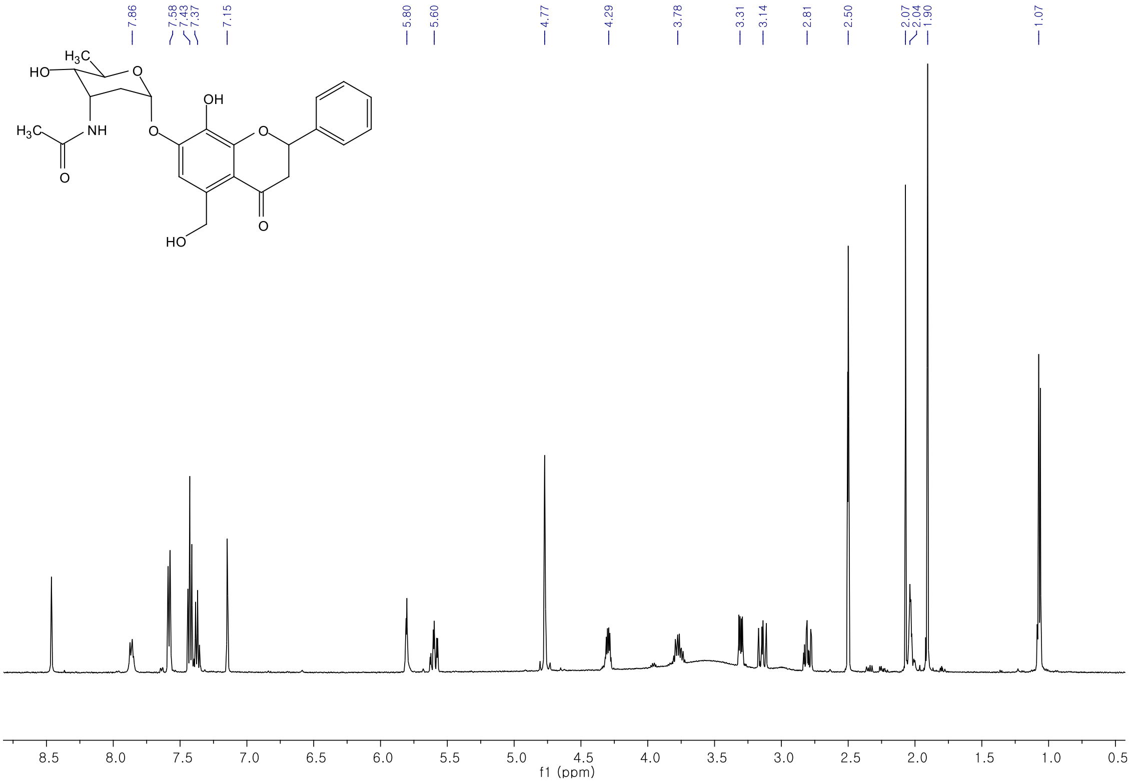

Figure S8. ^{1}H NMR spectrum of actinoflavoside C (**2**) at 600 MHz in DMSO.

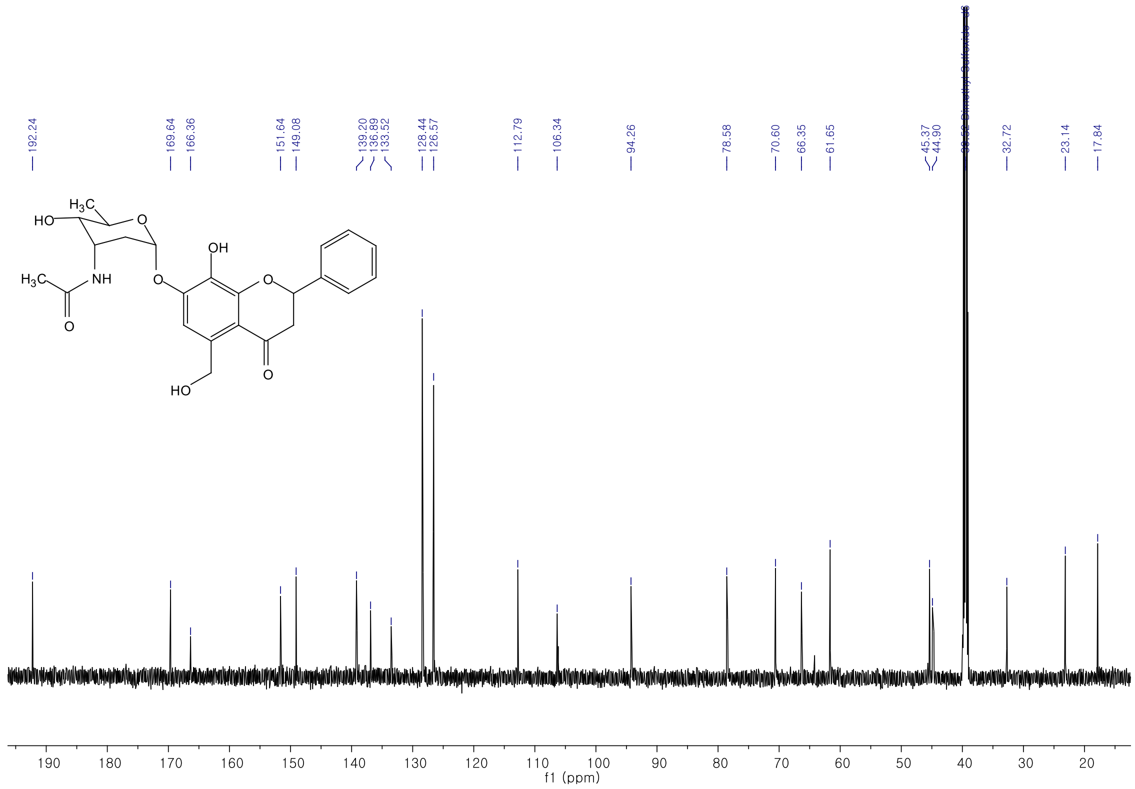

Figure S9. ^{13}C NMR spectrum of actinoflavoside C (**2**) at 150 MHz in DMSO.

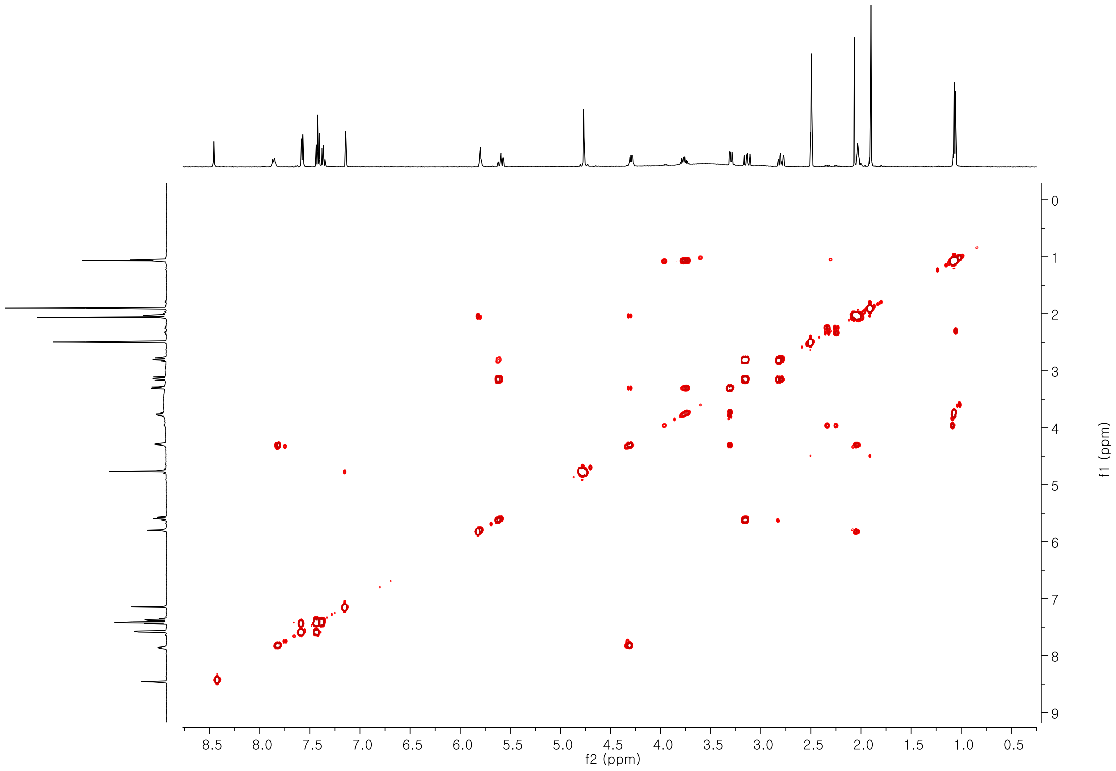

Figure S10. COSY NMR spectrum of actinoflavoside C (**2**) in DMSO.

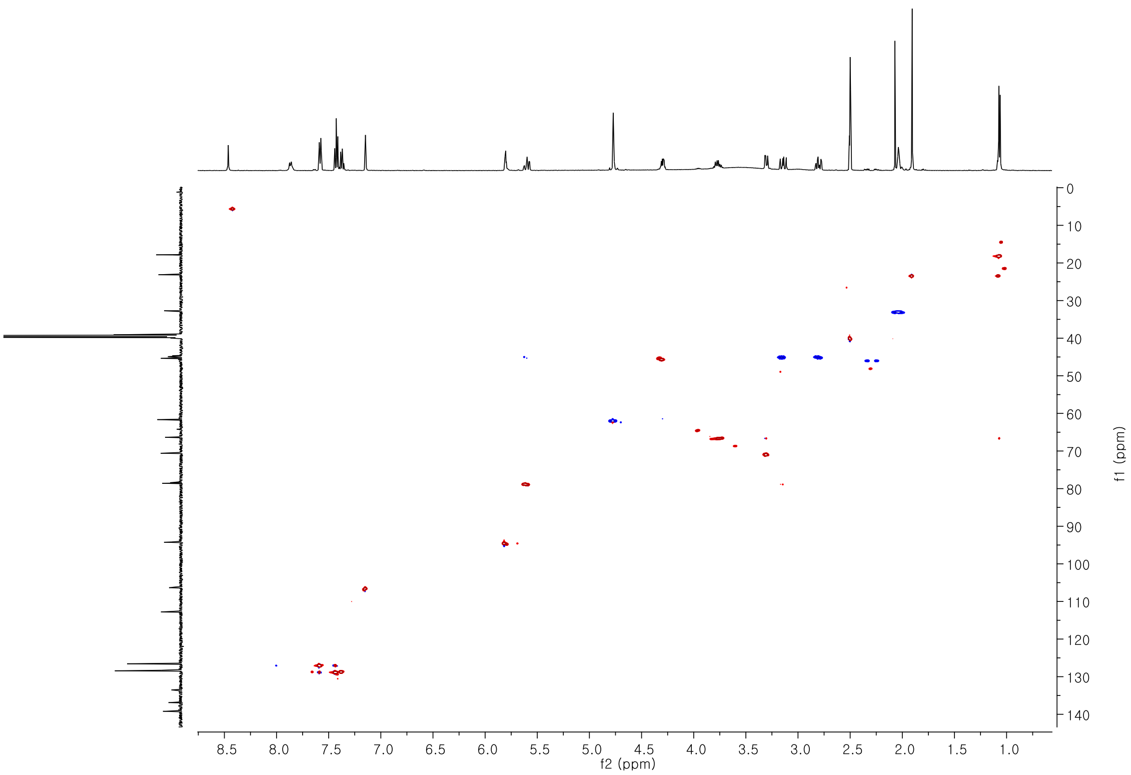

Figure S11. HSQC NMR spectrum of actinoflavoside C (**2**) in DMSO.

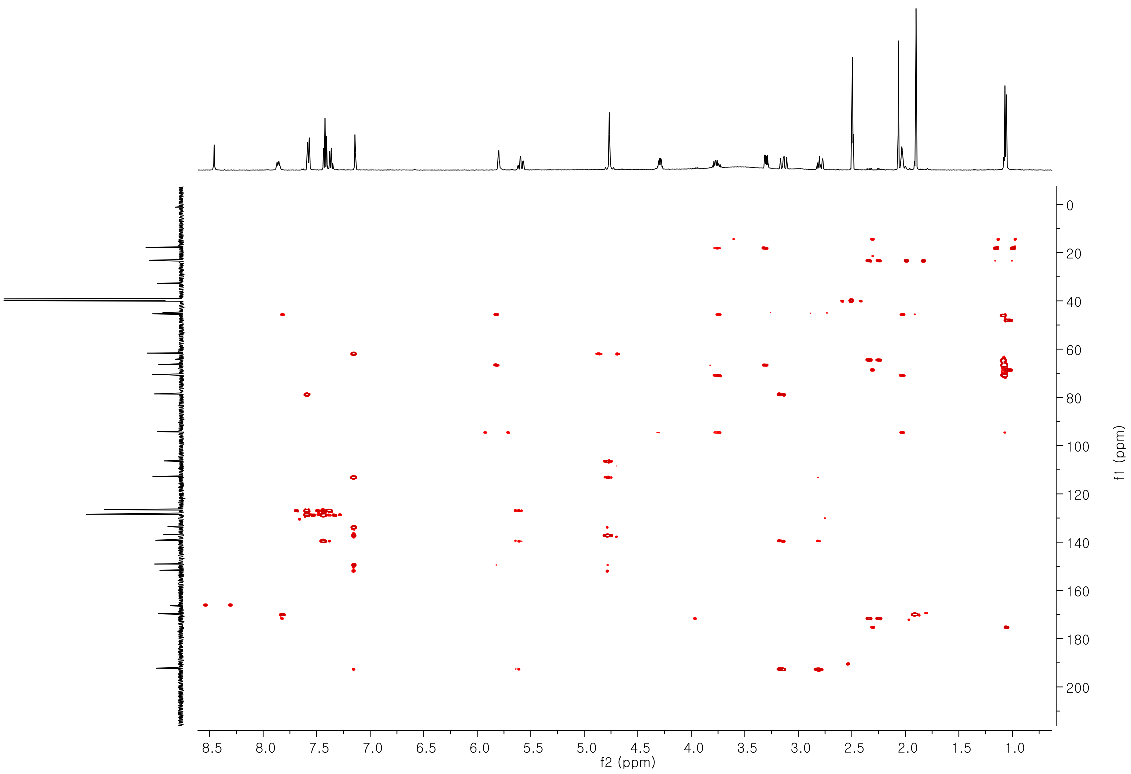

Figure S12. HMBC NMR spectrum of actinoflavoside C (**2**) in DMSO.

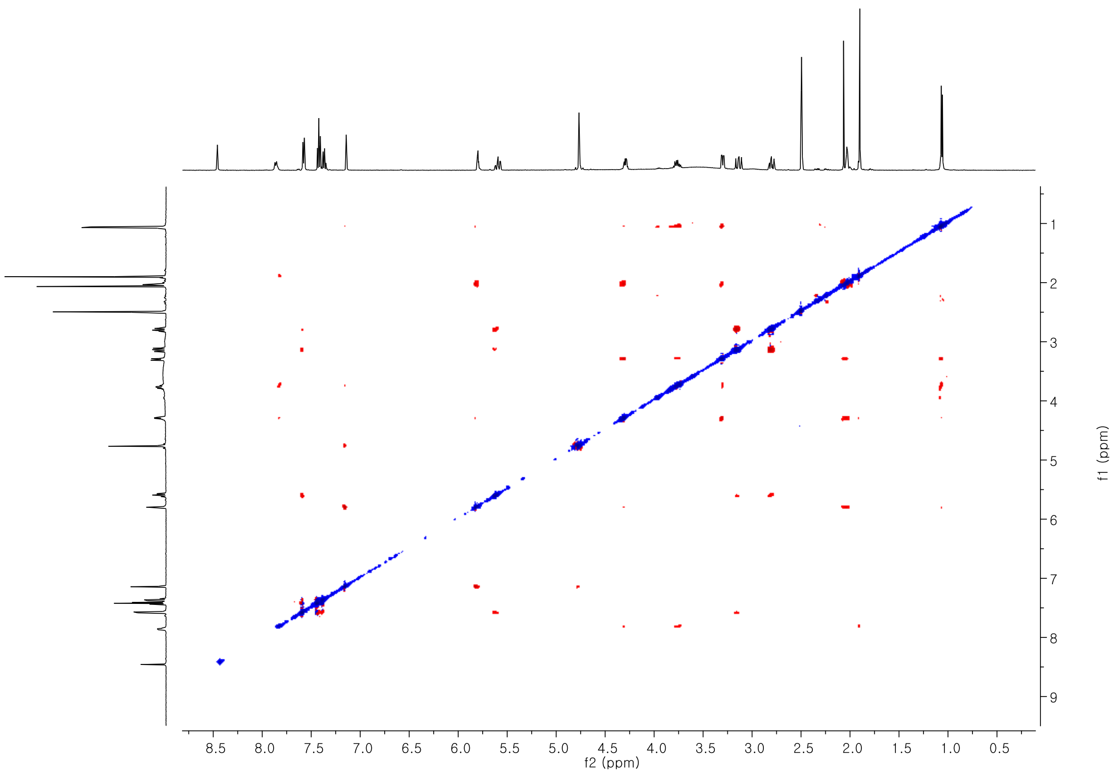

Figure S13. ROESY NMR spectrum of actinoflavoside C (**2**) in DMSO.

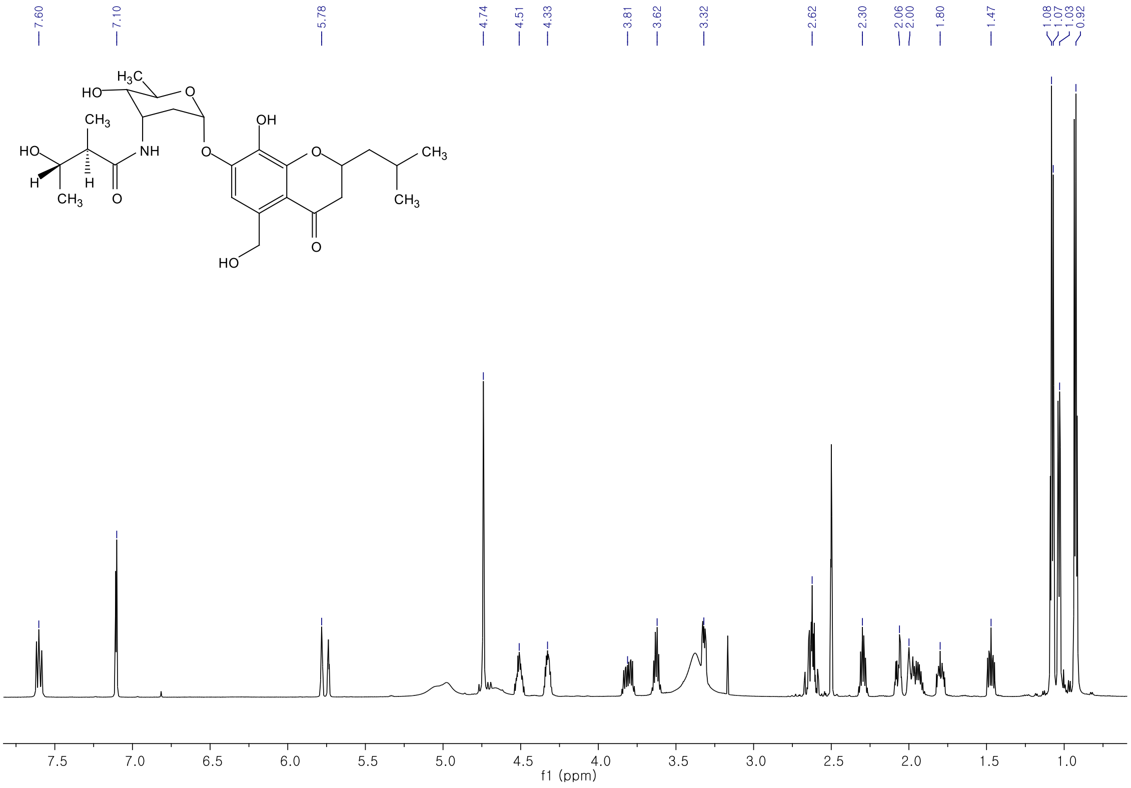

Figure S14. ^{1}H NMR spectrum of actinoflavoside D (**3**) at 600 MHz in DMSO.

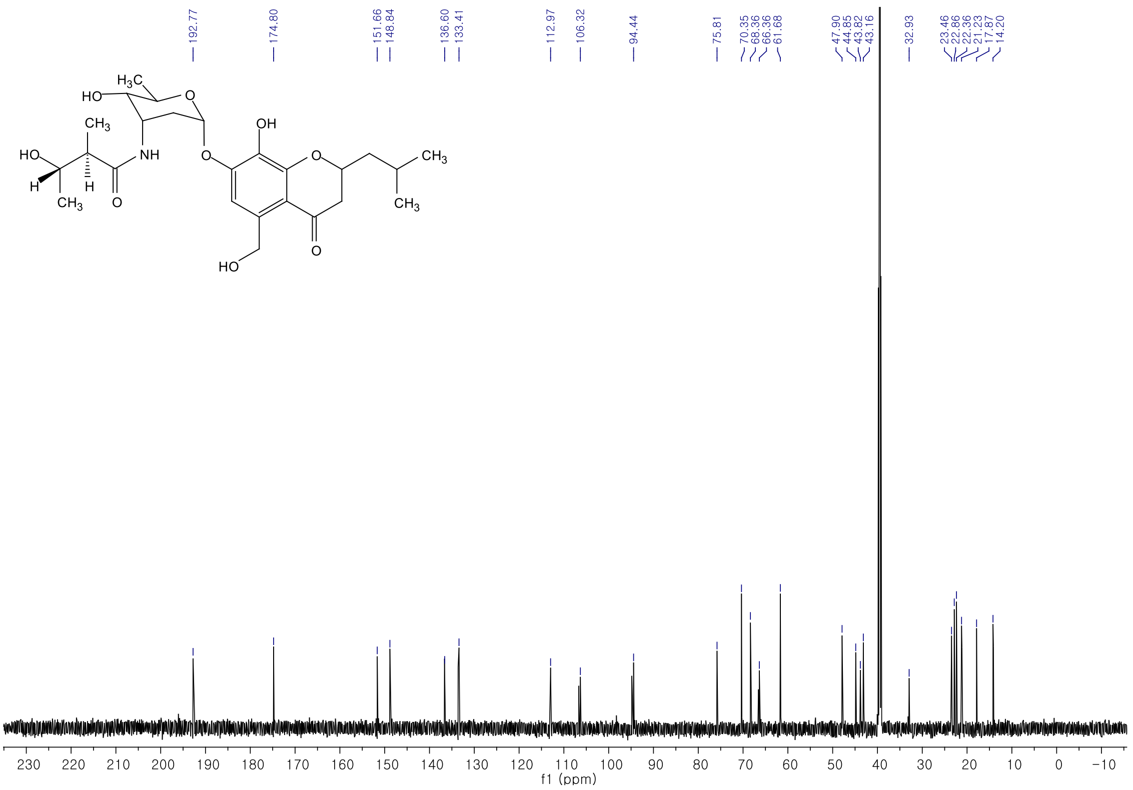

Figure S15. ^{13}C NMR spectrum of actinoflavoside D (**3**) at 150 MHz in DMSO.

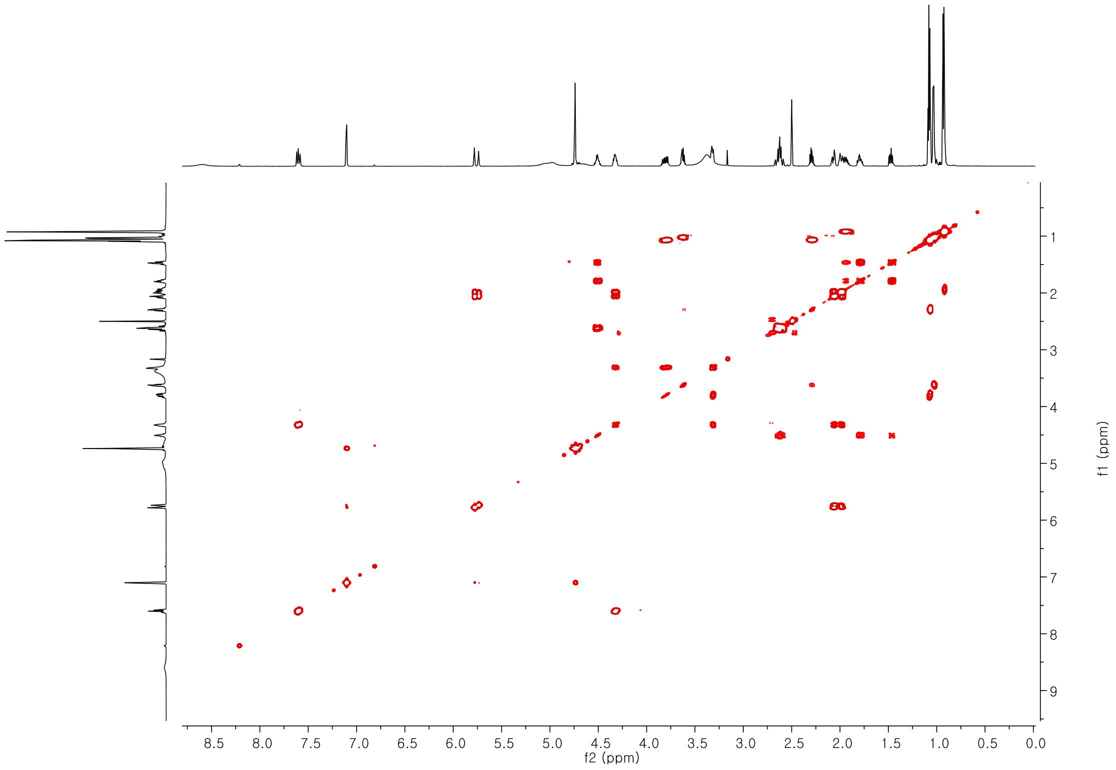

Figure S16. COSY NMR spectrum of actinoflavoside D (**3**) in DMSO.

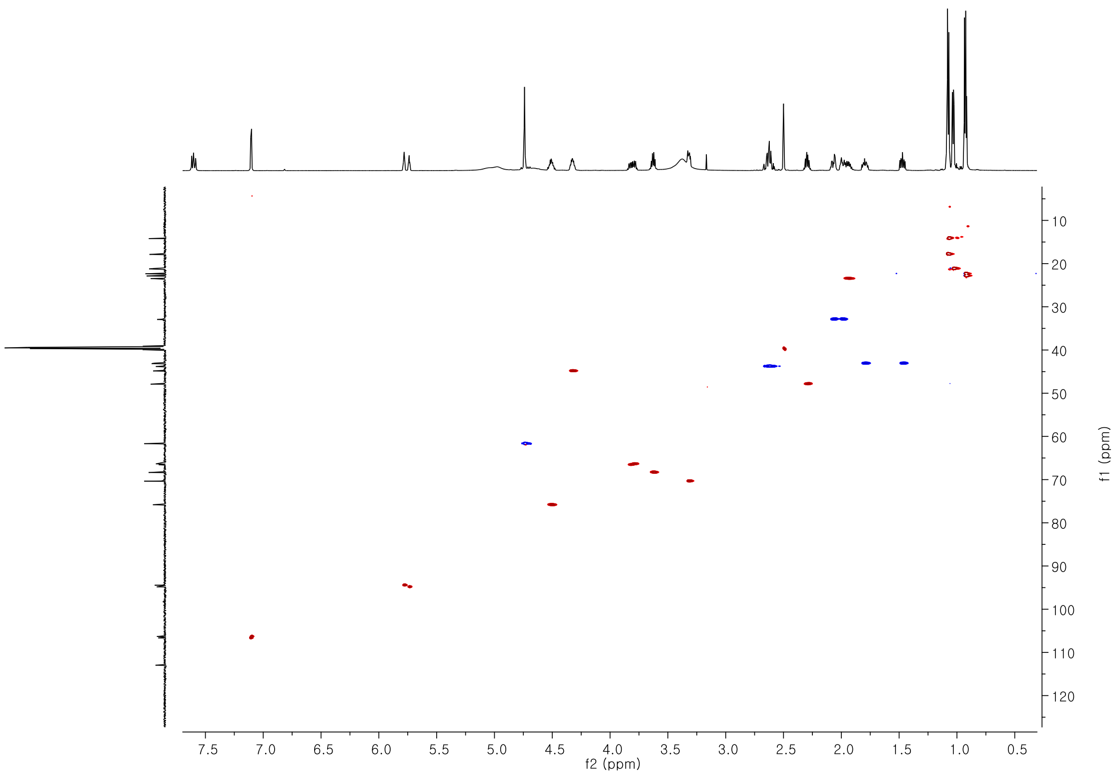

Figure S17. HSQC NMR spectrum of actinoflavoside D (**3**) in DMSO.

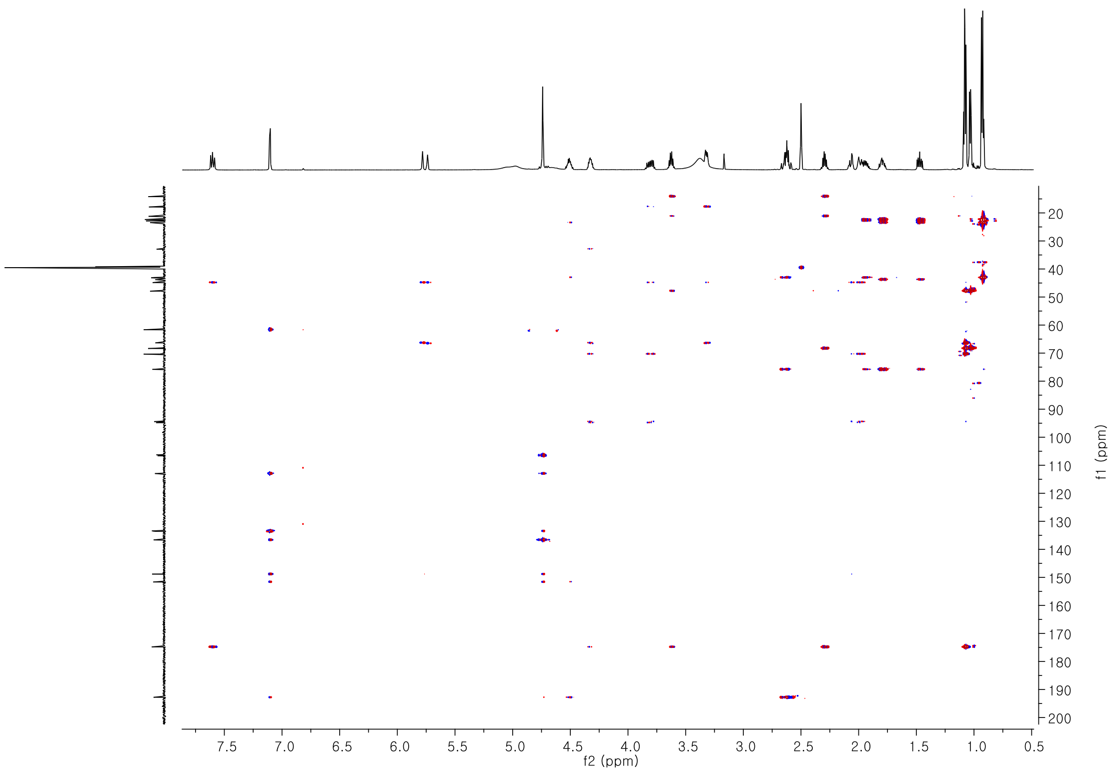

Figure S18. HMBC NMR spectrum of actinoflavoside D (**3**) in DMSO.

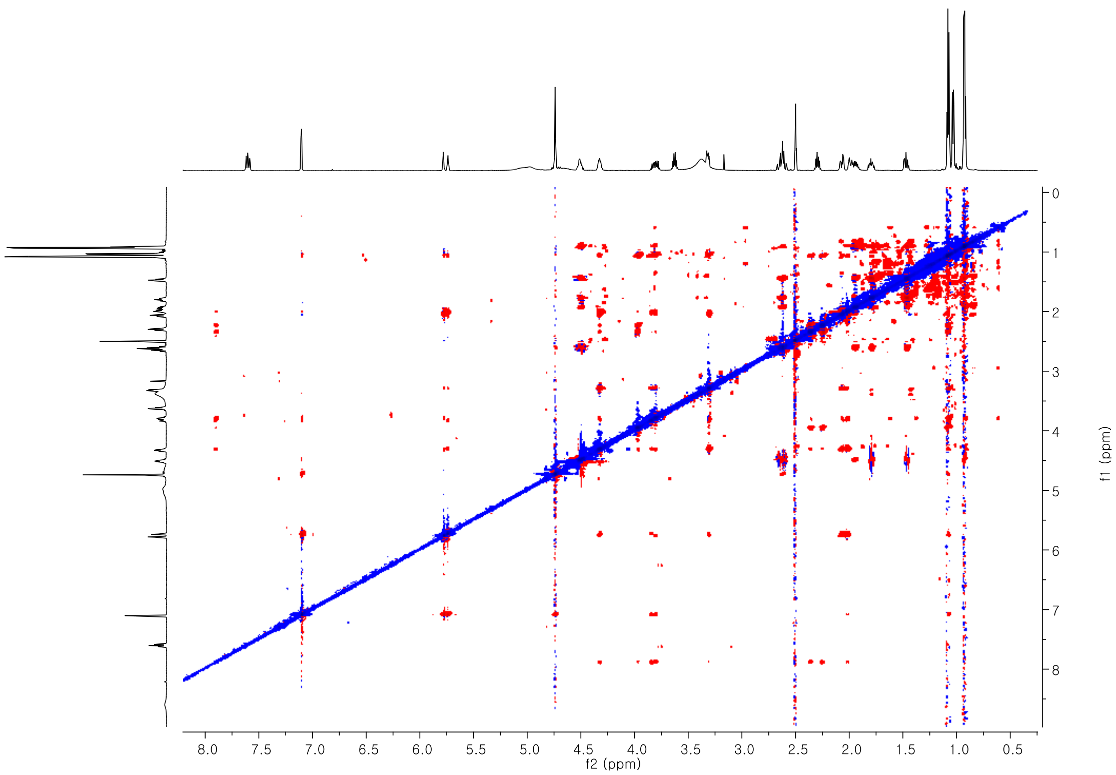

Figure S19. ROESY NMR spectrum of actinoflavoside D (**3**) in DMSO.

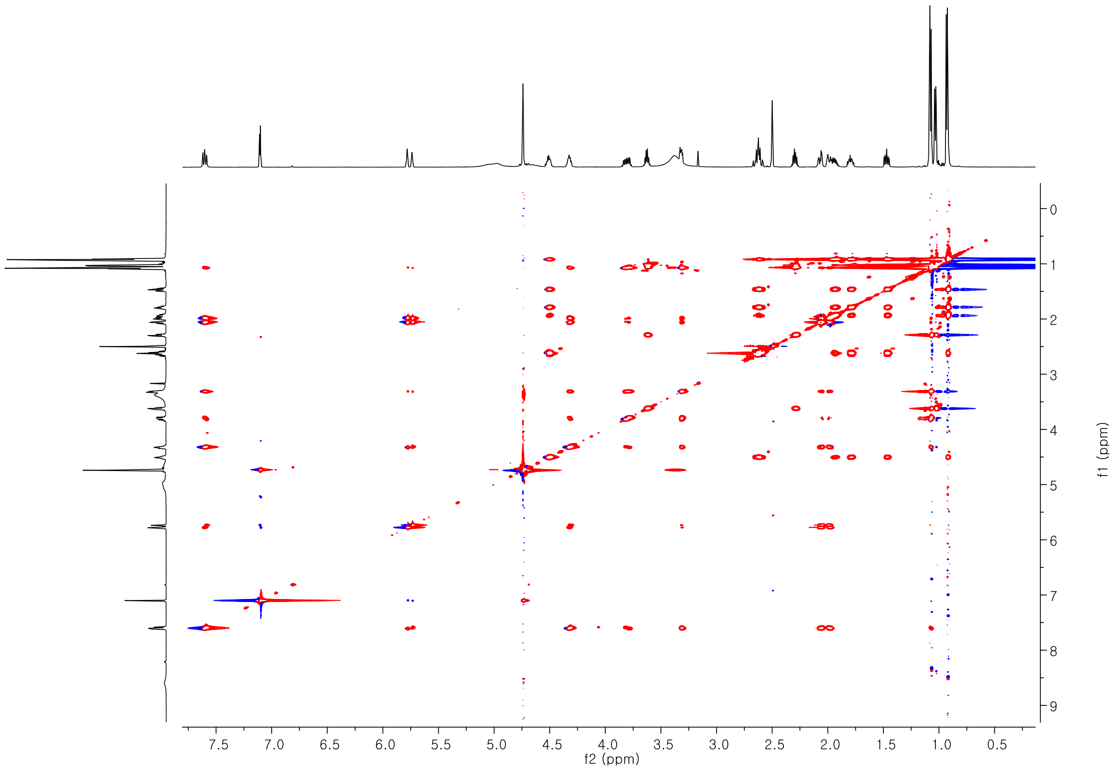

Figure S20. TOCSY NMR spectrum of actinoflavoside D (**3**) in DMSO.

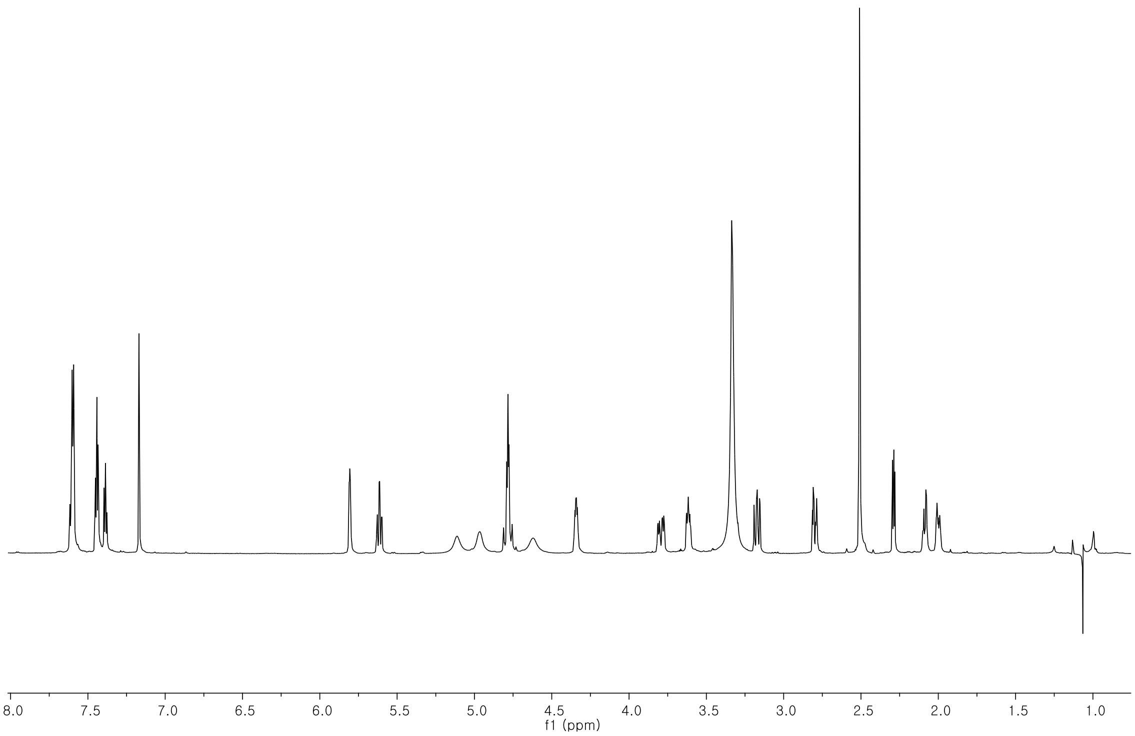

Figure S21. Methyl decoupling ^{1}H NMR spectrum of actinoflavoside B (**1**) at 800 MHz in DMSO.

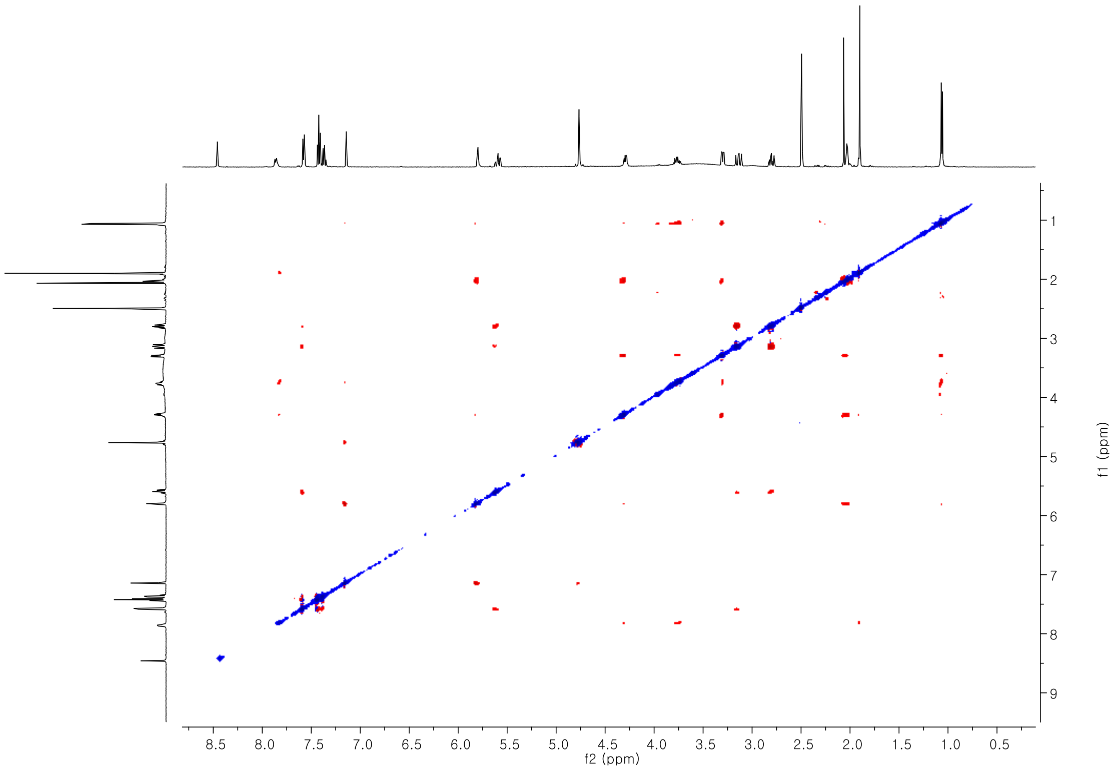

Figure S22. DQF-COSY NMR spectrum of actinoflavoside B (**1**) at 800 MHz in DMSO.

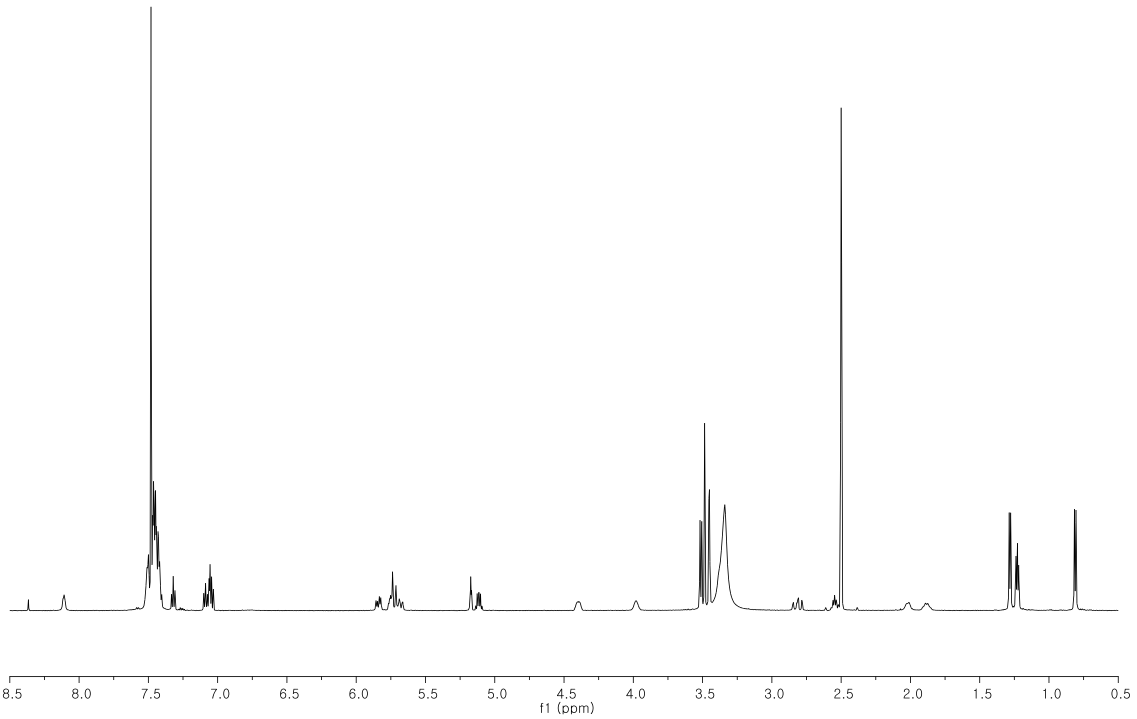

Figure S23. ^{1}H NMR spectrum of S-MTPA ester (**1a**) for actinoflavoside B (**1**) at 600 MHz in DMSO.

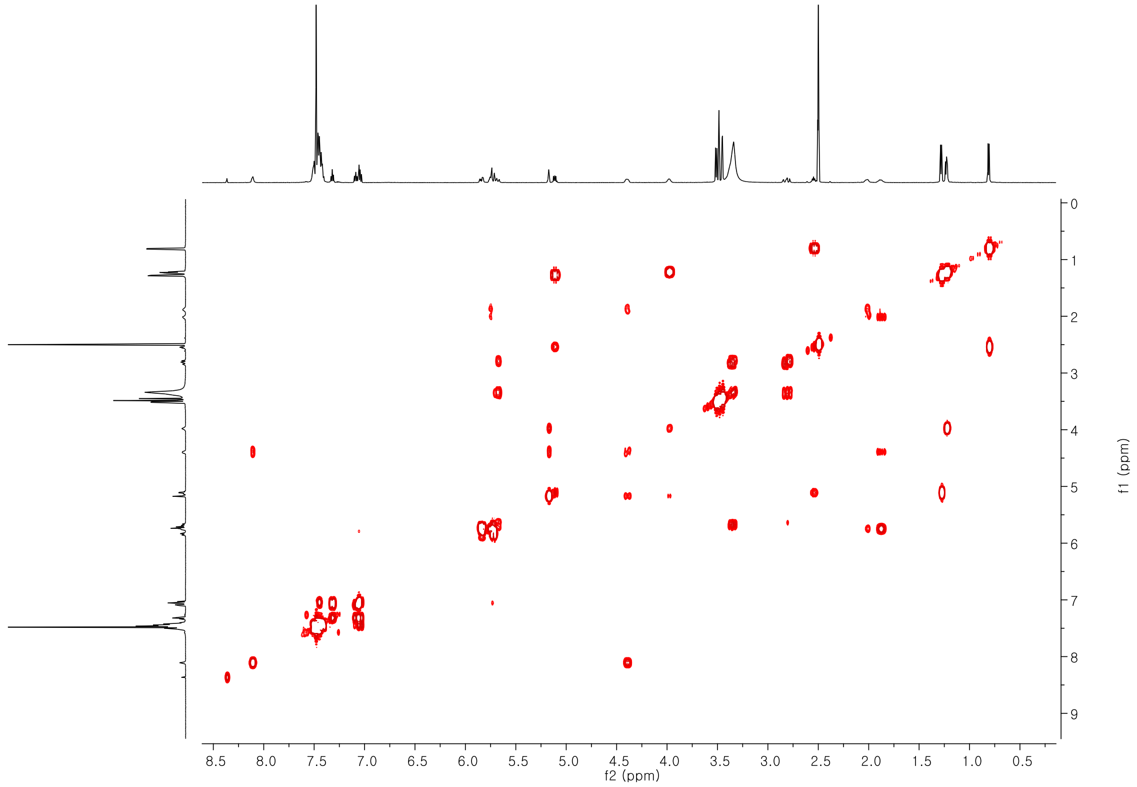

Figure S24. COSY NMR spectrum of S-MTPA ester (**1a**) for actinoflavoside B (**1**) at 600 MHz in DMSO.

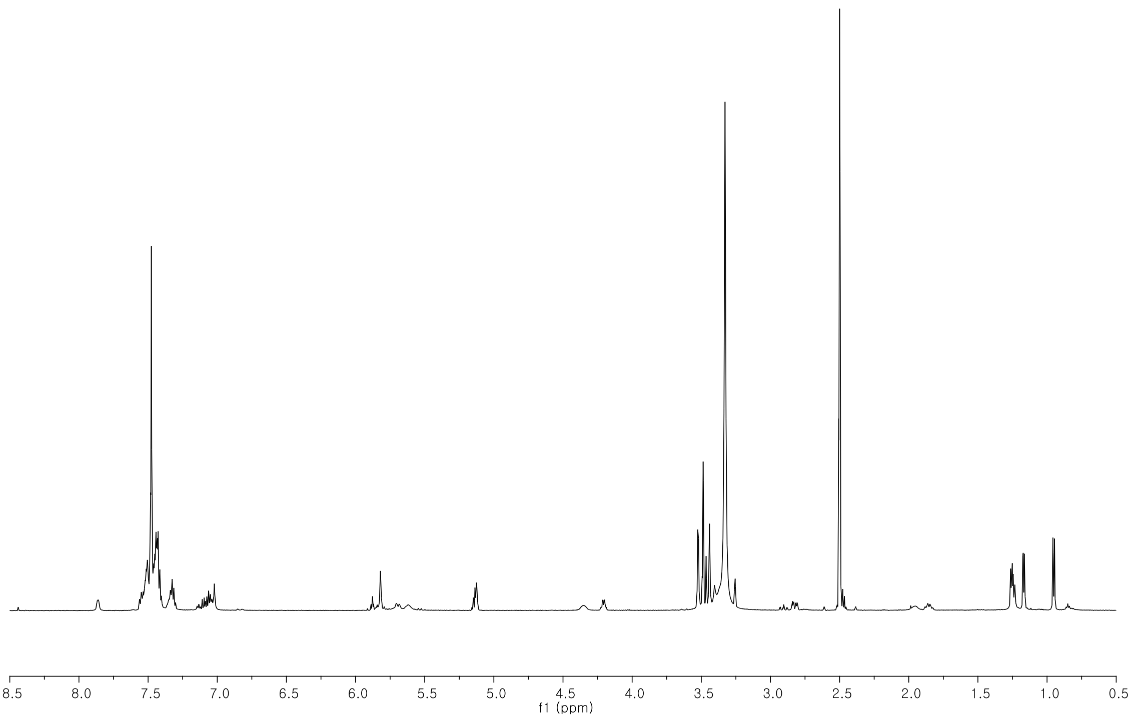

Figure S25. ^{1}H NMR spectrum of R-MTPA ester (**1b**) for actinoflavoside B (**1**) at 600 MHz in DMSO.

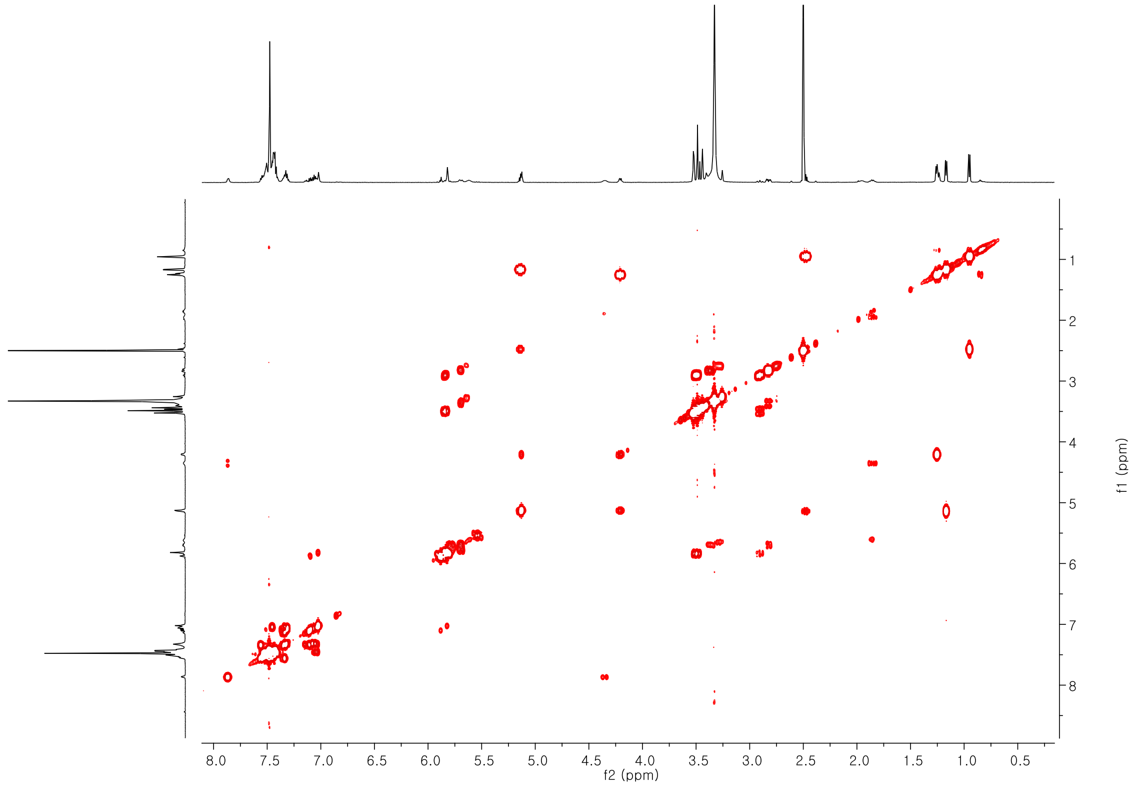

Figure S26. COSY NMR spectrum of R-MTPA ester (**1b**) for actinoflavoside B (**1**) at 600 MHz in DMSO.

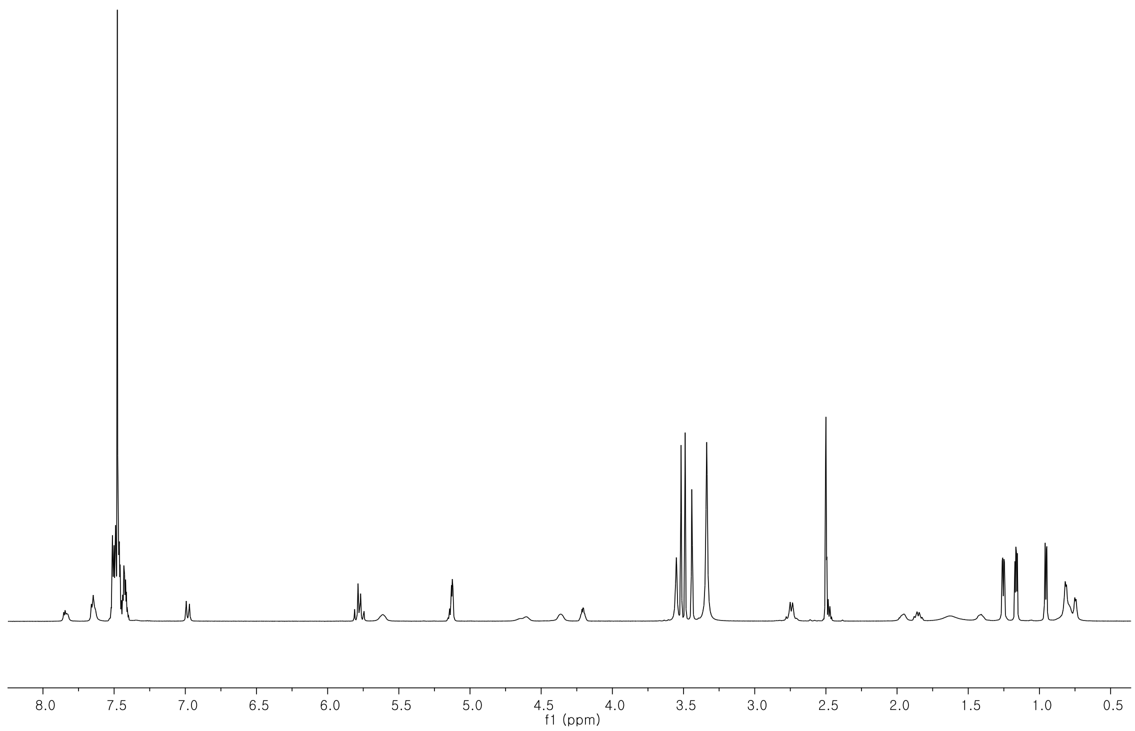

Figure S27. ^{1}H NMR spectrum of S-MTPA ester (**3a**) for actinoflavoside D (**3**) at 600 MHz in DMSO.

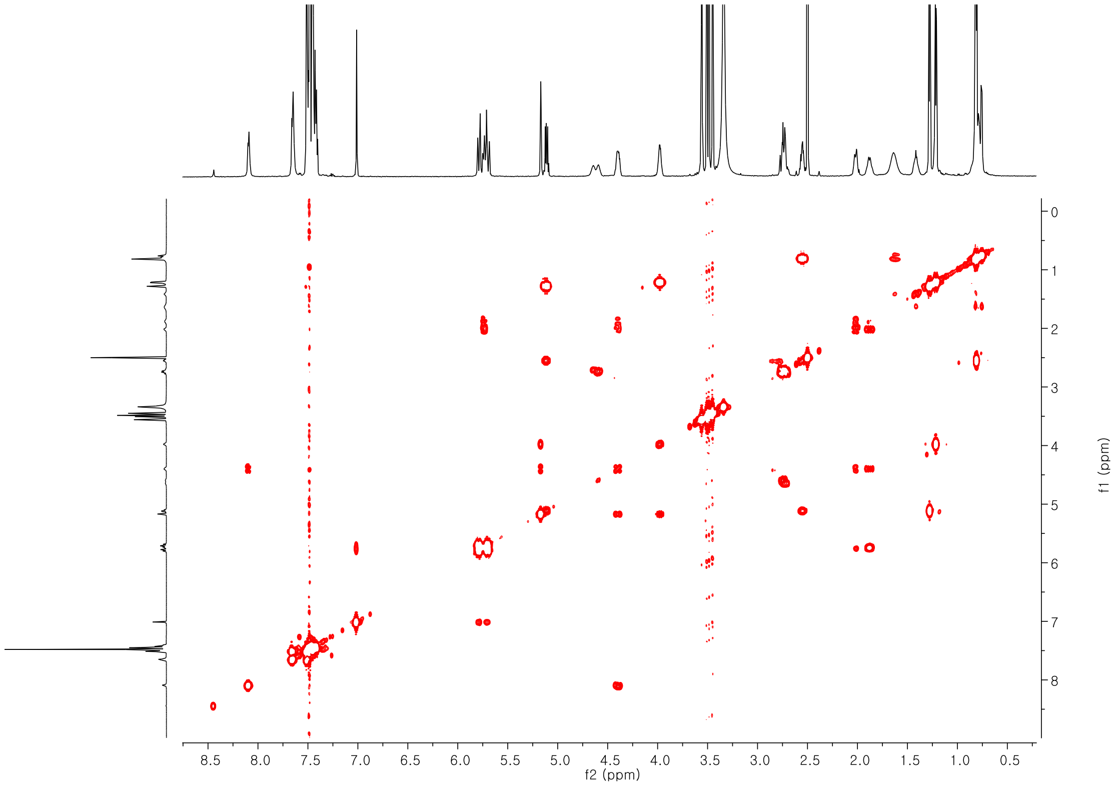

Figure S28. COSY NMR spectrum of S-MTPA ester (**3a**) for actinoflavoside D (**3**) at 600 MHz in DMSO.

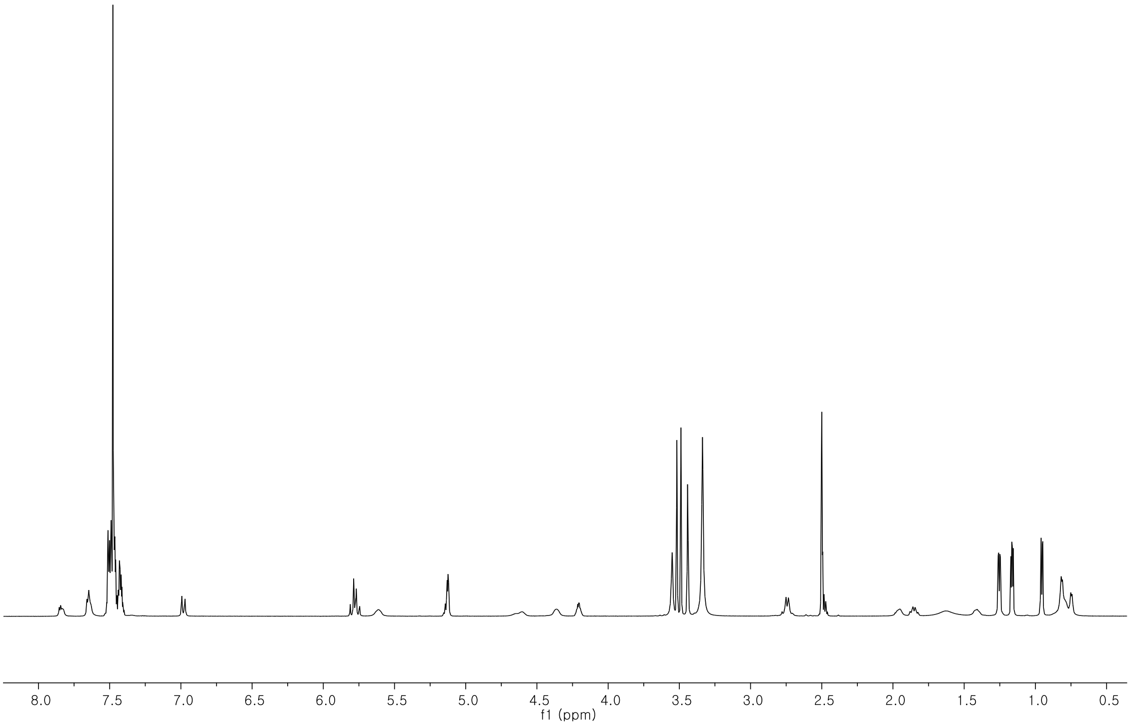

Figure S29. ^{1}H NMR spectrum of R-MTPA ester (**3b**) for actinoflavoside D (**3**) at 600 MHz in DMSO.

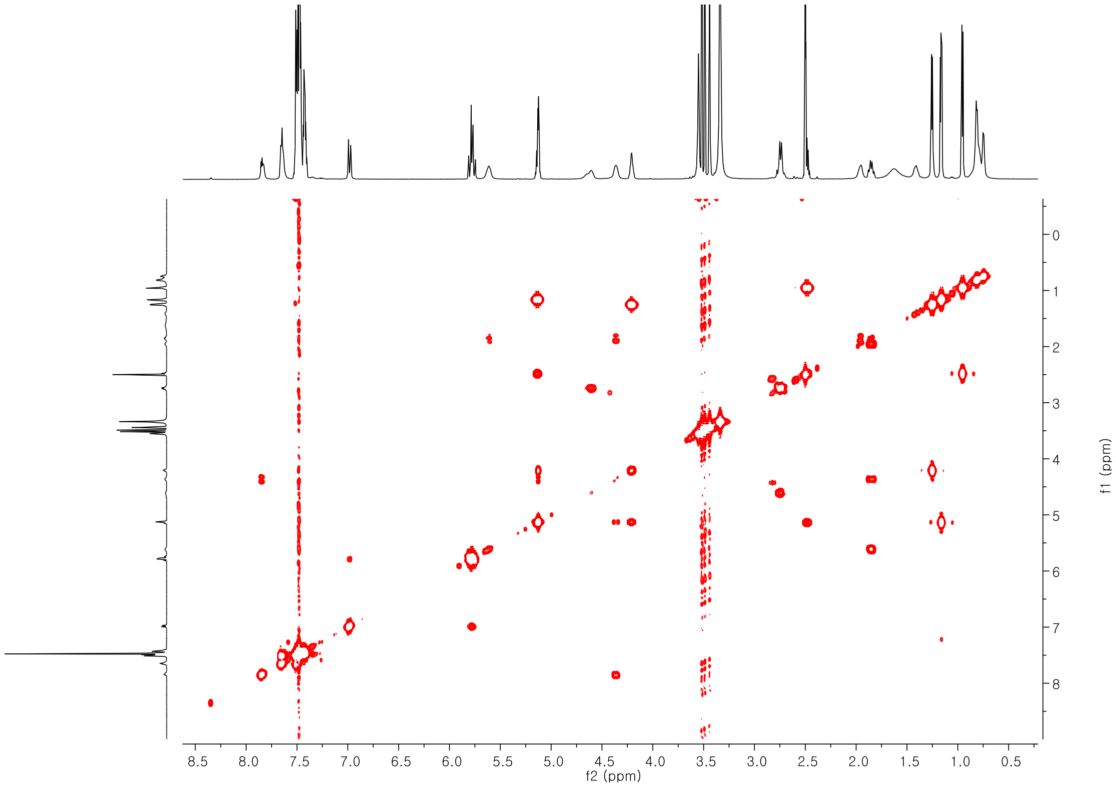

Figure S30. COSY NMR spectrum of R-MTPA ester (**3a**) for actinoflavoside D (**3**) at 600 MHz in DMSO.

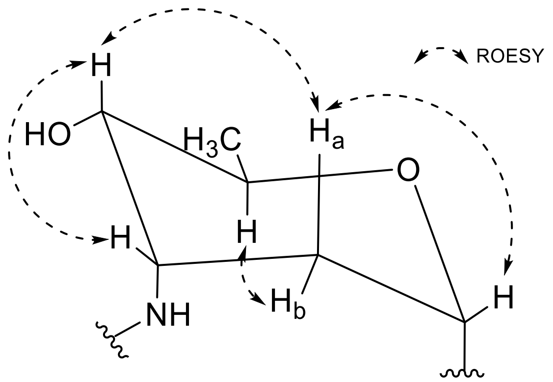

Figure S31. Key ROESY correlations of the 2, 3, 6-trideoxyaminosugar of acitnoflavoside B (**1**).

GCAGTGGGTATCGAGGCGGGGCACTTAATGCGTTAGCTGCGGCACGGACGACGTGGAATGTCGCCCACAC
CTAGTGCCCACCGTTTACGGCGTGGACTACCAGGGTATCTAATCCTGTTCGCTCCCCACGCTTTCGCTCC
TCAGCGTCAGTATCGGCCCAGAGATCCGCCTTCGCCACCGGTGTTCCTCCTGATATCTGCGCATTTCACC
GCTACACCAGGAATTCCGATCTCCCCTACCGAACTCTAGCCTGCCCGTATCGACTGCAGACCCGGGGTTA
AGCCCCGGGCTTTCACAACCGACGTGACAAGCCGCCTACGAGCTCTTTACGCCCAATAATTCCGGACAAC
GCTCGCGCCCTACGTATTACCGCGGCTGCTGGCACGTAGTTAGCCGGCGCTTCTTCTGCAGGTACCGTCA
CTTTCGCTTCTTCCCTGCTGAAAGAGGTTTACAACCCGAAGGCCGTCATCCCTCACGCGGCGTCGCTGCA
TCAGGCTTTCGCCCATTGTGCAATATTCCCCACTGCTGCCTCCCGTAGGAGTCTGGGCCGTGTCTCAGTC
CCAGTGTGGCCGGTCGCCCTCTCAGGCCGGCTACCCGTCGTCGCCTTGGTGAGCCATTACCTCACCAACA
AGCTGATAGGCCGCGGGCTCATCCTGCACCGCCGGAGCTTTCGAACCTCACAGATGCCCGTGAGGGTCAG
TATCCGGTATTAGACCCCGTTTCCAGGGCTTGTCCCAGAGTGCAGGGCAGATTGCCCACGTGTTACTCAC
CCGTTCGCCACTAATCCCCACCGAAGTGGTTCATCGTTCGACTTGCATGTGTTAAGCACGCCGCCAGCGT
TCGTCCTGAGCAGAAAAAAAAAACCTAAAAAACCGTACAATCCGGCGGAACATGAGGCTTAATTCCCAGGC
AACGGCAAAAAACCTTAACCAAGGCTTGGAATACACCGGGAAAACCATAGAAAATAGTGCCCCCCCTTGG
GGCCGGGGACAAGGTGGTGCAAGGGTATCCCTCCCCCCCCTGGCCCGGAAAAATTGGGGGTAATTCCCTC
CCAAAGGGGCAACCCATGTGTCGCTGGTGTGCCCGCAAGACACTCTCTGTGTGGTGTGGGAGAGCTCTCG
AACACACCCCGCGGCGGAATCTCACAGGAAAAGGGTGGAGCGACCACTCAATCCACATCCACCCCCCCCT
CTTATTGGGGGGGGGGAAAT

Figure S32. 16S rDNA sequence data of JML48.

GGGGGGCGACGTTGGGACTAGGTGTTGGCGACATTCCACGTCGTCGGTGCCGCAGCTAACGCATTAAGTT

CCCCGCCTGGGGAGTACGGCCGCAAGGCTAAAACTCAAAGGAATTGACGGGGGCCCGCACAAGCAGCGGA

GCATGTGGCTTAATTCGACGCAACGCGAAGAACCTTACCAAGGCTTGACATACACCGGAAAGCATCAGAG

ATGGTGCCCCCCTTGTGGTCGGTGTACAGGTGGTGCATGGCTGTCGTCAGCTCGTGTCGTGAGATGTTGG

GTTAAGTCCCGCAACGAGCGCAACCCTTGTTCTGTGTTGCCAGCATGCCCTTCGGGGTGATGGGGACTCA

CAGGAGACTGCCGGGGTCAACTCGGAGGAAGGTGGGGACGACGTCAAGTCATCATGCCCCTTATGTCTTG

GGCTGCACACGTGCTACAATGGCCGGTACAATGAGCTGCGATGCCGCGAGGCGGAGCGAATCTCAAAAAG

CCGGTCTCAGTTCGGATTGGGGTCTGCAACTCGACCCCATGAAGTCGGAGTTGCTAGTAATCGCAGATCA

GCATTGCTGCGGTGAATACGTTCCCGGGCCTTGTACACACCGCCCGTCACGTCACGAAAGTCGGTAACAC

CCGAAGCCGGTGGCCCAACCCCTTGTGGGAGGGAGCTGTCGAAGGTGGGACTGGCGATTGGGACGAAGTC

GTAACAAGGTAGCCGTACCGGAAGGTGCGGCTGGATCCCCCCCCCTTTTTATAAAAAAAAAAATTGGTTG

AGAAATTATTGTAGGGGAAATTCTGGGTCCAAGGTTCCCCTTTCCACACCGTTGTTTCCCCCCACATTGT

GGGCTATTTCGTCCGCGGCAAAGTATTCCAAATTTTCCCGAGCCCACTTTAAGCCGGGGCGAAAAAGGGG

GGGTAAACCCAGGGTTTAATCCCAGAGGTTTTTAAAGGGGAGTAGATATACCGCCCAAAAAACAATATTA

ATGGCCCCCCCATTTTTGGAAGAAAGATGTGCGCGCTCCTAATATGACTACCCCGCCCGCGGCGGGAAAT

AATCTTGGCTGGCTTCTCCTCCCGGGGAGGGGCCGTTTTTTTTTTTTTTCTTGCTGGGCAAGAAGTTCT

GTTTTTAGGAGGGAGAGGGGCCCCCCCACCCGAGCGGGGCGCCGTCCCTAACGATGAGGGGGGGGAGGG

AGAAGAAAAAAAATCTACACCACCCCCCCCCCCCCCTTCTCAGAGAGGGAGGGGGCGGGGGGGGCGGGT

TGTACCAATTGGGGGGTGGAGGGAGCCCTCTCCTCCCCTCCACGGGGCGCGCCCCCCCTCCCTCCCTAGT

GGTGTGTGTGGTGCTTTCTCAACGCAAAGGGGGGGGGCGGCGCCCCCCCCCTACCACATAGTTGCTCCTA

TCTCTCTCCCCTAGGAGGAGGAGAGGAAGTATAAAAACTTCCTCCCCCCCCCCCCCCCGGGGGAAAAAA

Figure S33. 16S rDNA sequence data of JMS33.

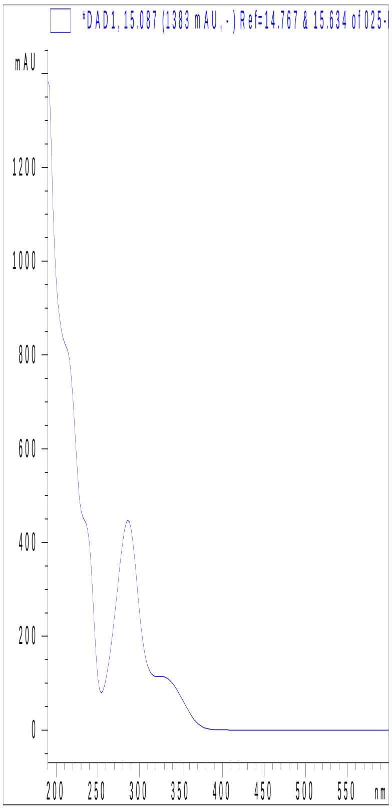

(1)

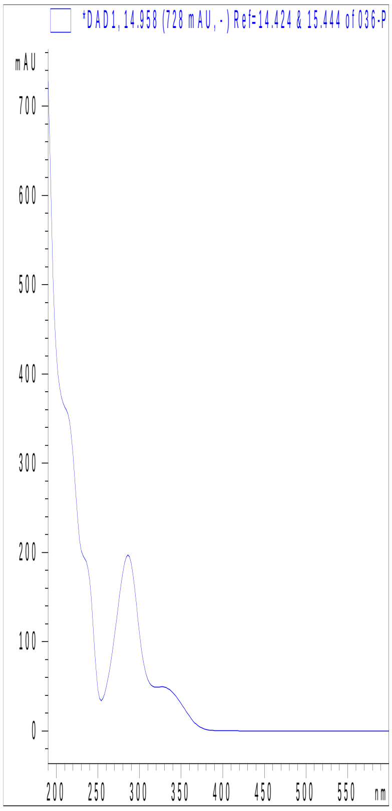

(2)

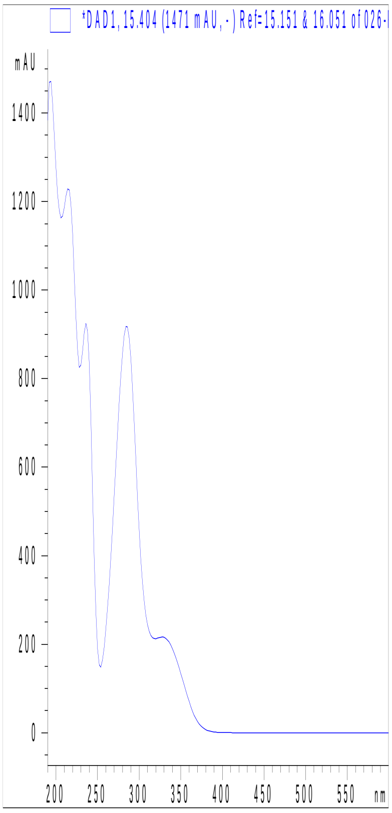

(3)

Figure S34. UV spectrum of actinoflavoside B-D (**1-3**).

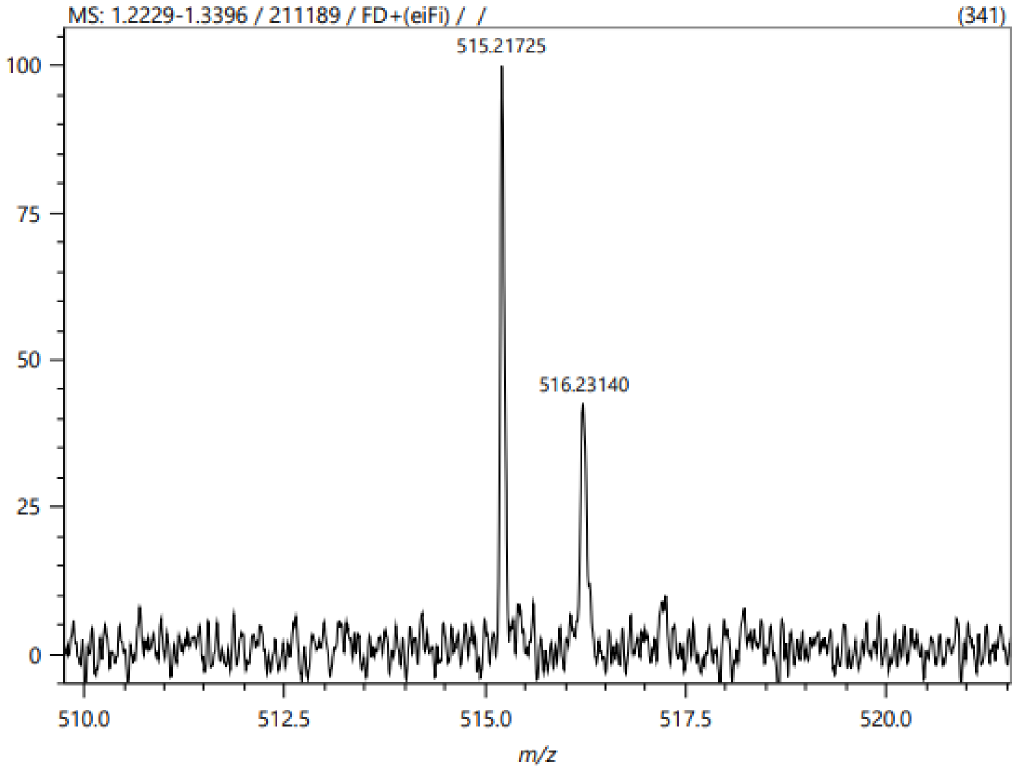

Figure S35. HR-TOF-MS data of actinoflavosdie B (**1**).

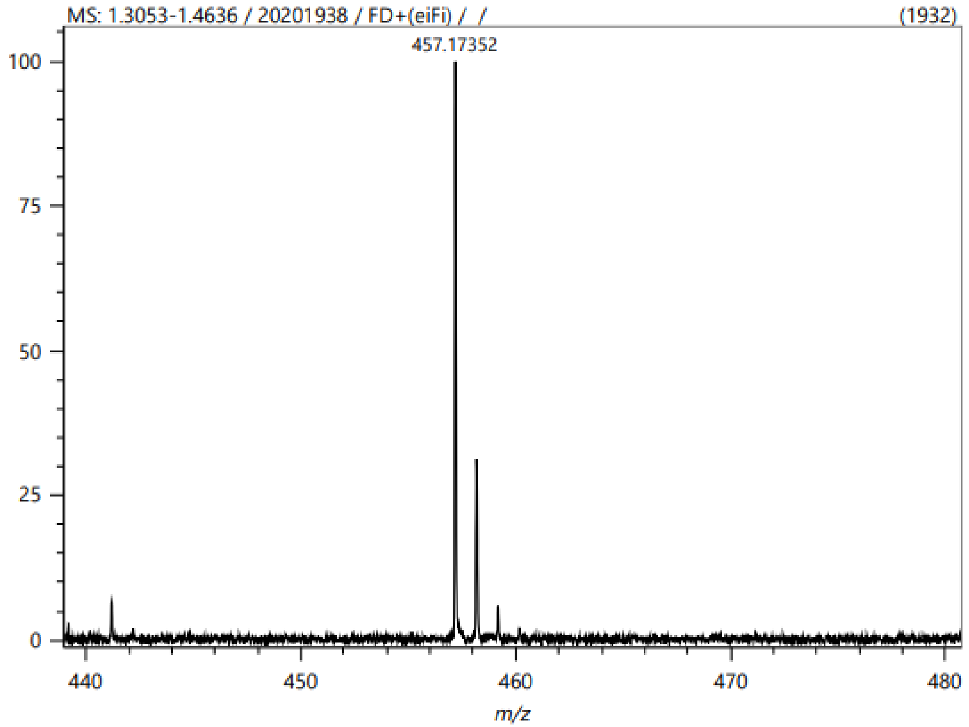

Figure S36. HR-TOF-MS data of actinoflavosdie C (**2**).

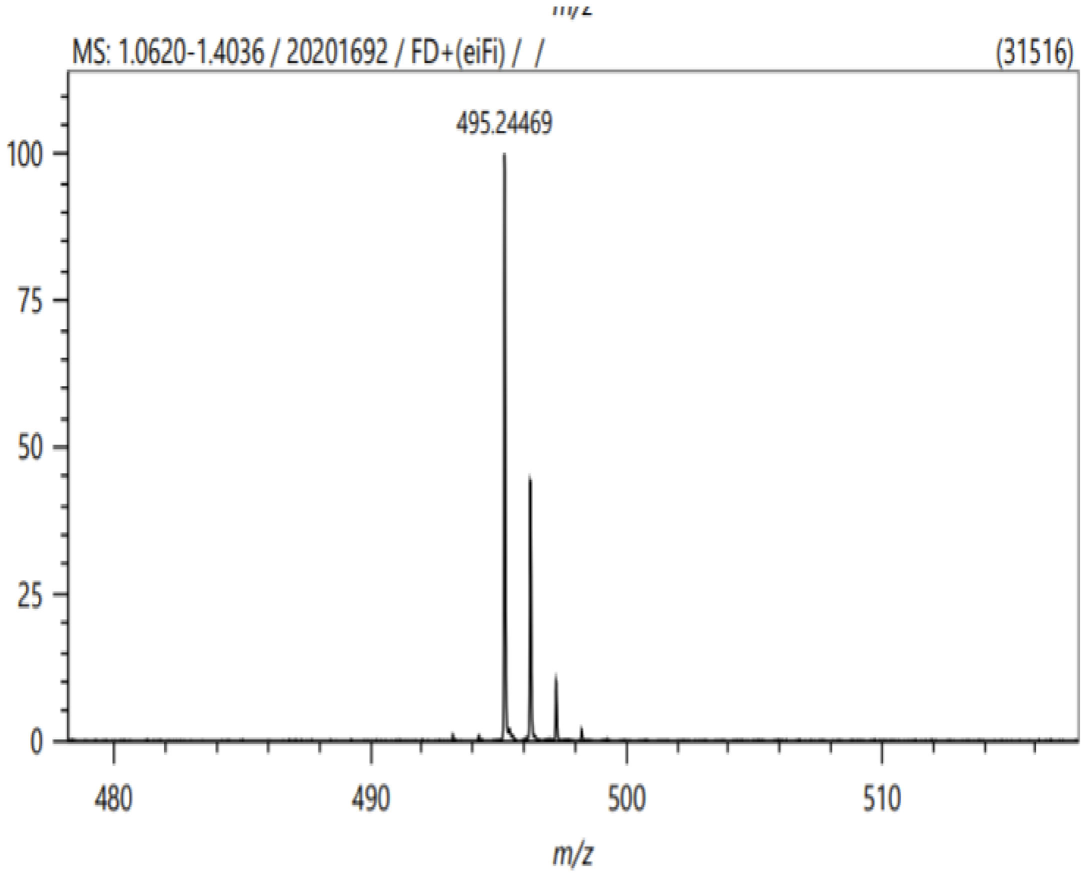

Figure S37. HR-TOF-MS data of actinoflavosdie D (**3**).

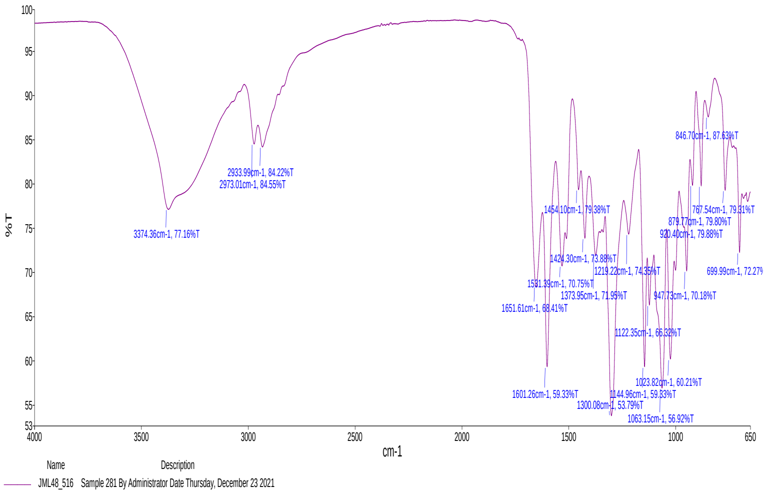

Figure S38. IR spectrum of actinoflavosdie B (**1**).

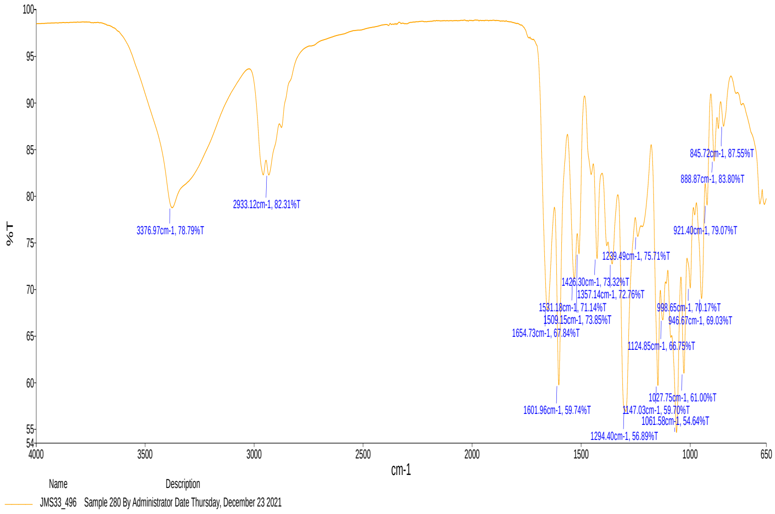

Figure S39. IR spectrum of actinoflavosdie C (**2**).

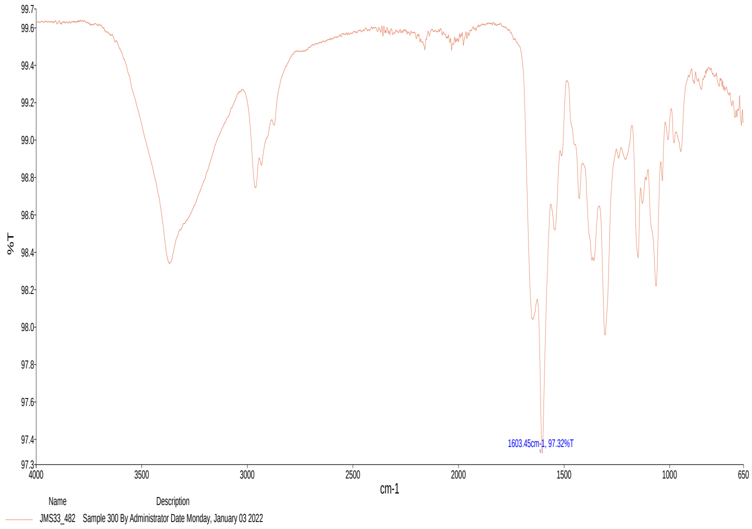

Figure S40. IR spectrum of actinoflavosdie D (**3**).

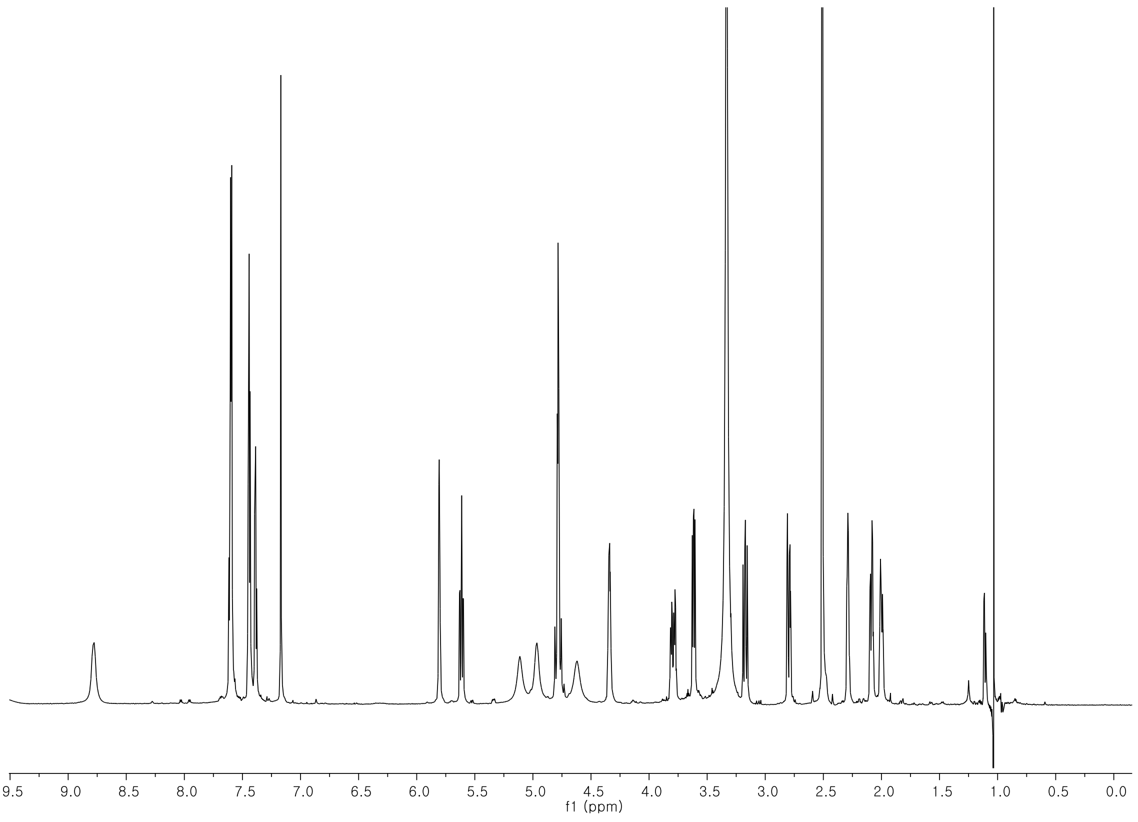

Figure S41. H-4′′′ decoupling ^{1}H NMR spectrum (800 MHz) of actinoflavoside B (**1**) in DMSO.

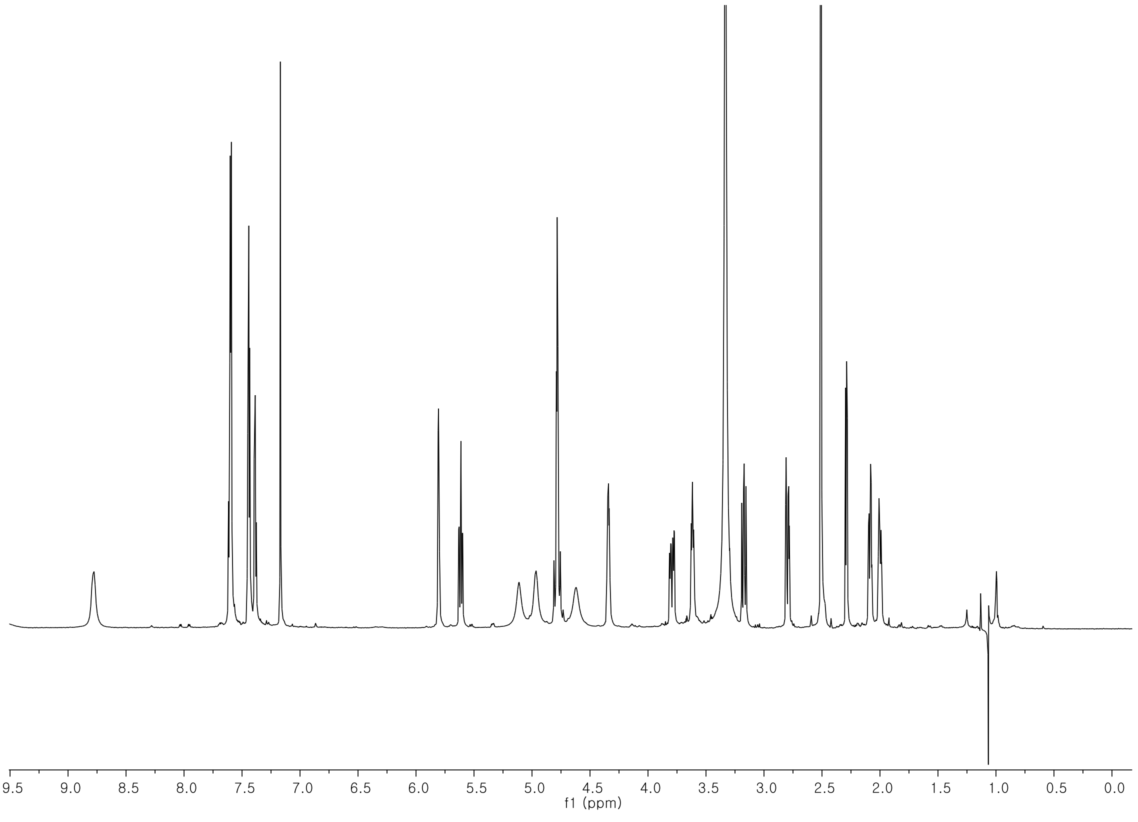

Figure S42. H-5′′′ decoupling ^{1}H NMR spectrum (800 MHz) of actinoflavoside B (**1**) in DMSO.

Compound	MIC (mM)			
	**Gram (+) Bacteria**		**Gram (-) negative**	
	***B. subtilis* ATCC 6051**	***P. aeruginosa* KCTC 22073**	***E. coli* ATCC 11775**	***Er. rhapontici* ATCC 29283**
**1**	0.14	0.29	-	-
**2**	-	-	-	-
**3**	-	0.3	-	-
Gentamicin	0.42	0.42	0.42	0.42

Table S1. Minimum inhibitory concentration (MIC) of **1–3** against Gram-positive and Gram-negative bacterial strains.
